# Differences in health outcomes of orthopedic rehabilitation after hip or knee replacement: a prospective pilot study benchmarking 23 rehabilitation facilities using Patient-Reported Outcome Measures (PROMs)

**DOI:** 10.1186/s42836-026-00401-x

**Published:** 2026-08-03

**Authors:** Volker Beierlein, Ralf Bürgy, Torsten Kirsch, Johannes Kneißl, Matthias Köhler, Gert Krischak, Gina Sophie Labahn, Annabelle Neudam, Holger Schulz

**Affiliations:** 1 Department of Medical Psychology, University Medical Center Hamburg-Eppendorf, Martinistraße 52, Hamburg, 20246 Germany; 2 MediClin AG, Okenstraße 27, Offenburg, 77652 Germany; 3 VITREA Holding Deutschland GmbH, Seeuferweg 10, Damp, 24351 Germany; 4 Medical Park SE, Freiberger-Platz 1, Amerang, 83123 Germany; 5 Nanz Medico GmbH & Co. KG, Wilhelmsplatz 11, Stuttgart, 70182 Germany; 6 4QD-Qualitätskliniken.de GmbH, Friedrichstraße 60, Berlin, 10117 Germany

**Keywords:** Hip arthroplasty, Knee arthroplasty, Rehabilitation, Benchmark, ProQI

## Abstract

**Background:**

Patient-reported outcome measures (PROMs) are increasingly used for benchmarking hip and knee arthroplasty, yet evidence on their feasibility for public reporting of short-term rehabilitation outcomes remains limited. International initiatives such as ICHOM recommend combining disease-specific and generic PROMs, but practical frameworks for risk-adjusted benchmarking in routine rehabilitation are scarce.

**Methods:**

In this prospective pilot study, 23 German rehabilitation facilities contributed data on patients following total hip (THA) or knee arthroplasty (TKA). Patients completed the HOOS-PS or KOOS-PS and the VR-12 PCS/MCS at admission and discharge after a standardized three-week multimodal rehabilitation program. For primary analyses, 961 THA and 961 TKA cases with complete pre- and post-data on at least one PROM were available. Facility-level unadjusted change was quantified using Cohen’s d_z_. Risk adjustment used pooled multiple linear regression including baseline PROMs, age, sex, and additional covariates retained via backward elimination (*p* < 0.10), yielding standardized residual effect sizes (d_res_) per facility. A composite Patient-Reported Outcome Quality Index (ProQI) weighted disease-specific standardized residuals at 50% and VR-12 PCS/MCS at 25% each, rescaled to a 0–100 metric with a normative mean of 90 and SD 30.

**Results:**

Pooled pre-post effect sizes indicated clinically relevant improvements (THA: d_z_ = 0.76 HOOS-PS, 0.76 PCS, 0.45 MCS; TKA: 0.83 KOOS-PS, 0.71 PCS, 0.39 MCS), with between-facility variability exceeding discrimination thresholds. Risk-adjusted d_res_ identified facilities performing above or below expectations. ProQI values showed substantial dispersion (THA range 75.4–100; TKA 76.9–100), enabling intuitive differentiation of providers. Overall, ProQI completeness was 69% (THA) and 72% (TKA). Non-completer analyses showed small baseline differences but no systematic ProQI bias.

**Conclusion:**

PROM-based, risk-adjusted benchmarking of short-term rehabilitation outcomes after hip and knee arthroplasty is feasible and meaningfully discriminative. The ProQI composite offers an interpretable, biopsychosocially grounded index for public reporting and quality management. Future work should enhance data completeness, refine risk adjustment, and validate the approach across broader cohorts and settings.

**Supplementary Information:**

The online version contains supplementary material available at 10.1186/s42836-026-00401-x.

## Background

In recent years, there has been an increase in the use of Patient-Reported Outcome Measures (PROMs), ranging from research and pilot projects to large-scale national implementations [1]. PROMs are standardized tools used to collect information directly from patients about their health conditions. They capture the patient’s perspective on their physical, mental, and social well-being, offering valuable insights that may not be apparent through clinical assessments alone. PROMs are applied in clinical trials, routine care, and health services research to evaluate the effectiveness of interventions and improve patient-centered care. Many PROMs focus on different dimensions of health-related quality of life (HRQoL), such as physical functioning, emotional well-being, social interactions, and pain. Some are designed as generic instruments to assess overall health status, while others are disease-specific tools tailored to particular conditions.

Examples of PROMs implementation in orthopedics can be found in Canada, where they serve as the national standard for evaluating hip and knee replacement outcomes [2, 3], and in the United States, where the Centers for Medicaid and Medicare Services (CMS) requires the use of both general and joint-specific PROMs to assess quality of care in total joint arthroplasty. A recent systematic review and meta-analysis identified 72 studies of adults undergoing total knee arthroplasty (TKA) that used self-reported function as a PROM [4]. Another systematic review on the efficacy of physiotherapy after total hip arthroplasty (THA) identified 20 studies [5]. Its key finding was that most studies carried a high risk of bias, limiting causal inferences. Furthermore, the evidence for the effectiveness of physiotherapy after total hip arthroplasty (THA) remains inconclusive due to heterogeneity in intervention characteristics, control conditions, follow-up durations, and outcome measures.

Beyond clinical evaluation, PROMs are increasingly utilized as key performance indicators for institutional benchmarking. However, using PROMs for comparative public reporting requires risk-adjustment models to account for heterogeneous patient populations (case-mix), ensuring that differences in outcomes reflect actual service quality rather than initial patient risk.

For patients undergoing THA or TKA, widely used disease-specific PROMs are the physical function short forms of the Hip Disability and Osteoarthritis Outcome Score (HOOS-PS) and the Knee Injury and Osteoarthritis Outcome Score (KOOS-PS). The HOOS-PS and KOOS-PS have been endorsed as appropriate outcome measures by international expert recommendations, such as the International Consortium for Health Outcome Measurement (ICHOM), as well as by arthroplasty registries and clinical research studies. Both instruments are valid and responsive for assessing physical function in THA and TKA [6–9]. In addition to disease-specific assessments, international recommendations emphasize the importance of generic questionnaires to evaluate changes in HRQoL across therapies [9]. The Veterans RAND 12-Item Health Survey (VR-12) is a generic instrument that includes both mental and physical health dimensions.

In Germany, inpatient or day-care rehabilitation following THA or TKA is standardized and typically lasts three weeks. Unlike in many other countries, rehabilitation in Germany following THA or TKA is multimodal rather than exclusively physiotherapy-based. According to analyses of therapeutic procedure data [10, 11], most patients receive physiotherapy (99%), physical therapy (96%), exercise therapy (89%), and health education (89%). More than half undergo occupational therapy or nursing interventions (61%), joint-specific education (51%), and continuous passive motion (CPM) therapy (51%). However, there is little evidence on the extent to which outcomes differ between rehabilitation facilities despite the broadly standardized multimodal approach. Although German rehabilitation follows standardized clinical pathways, the degree of institutional variation in outcomes remains largely unexamined. Identifying whether differences in PROMs are driven by facility-specific performance or by non-random patient allocation is a prerequisite for establishing a fair and transparent national reporting system.

The REHAPORTAL, a national web-based platform, provides patients, referring physicians, and statutory social and health insurance providers with transparent information on rehabilitation facilities and orientation through the presentation of achieved quality indicators. However, outcome quality assessed by pre-post measurements using PROMs has not yet been incorporated as a comparative performance metric. Against this background, the present study, a collaborative project between the Department of Medical Psychology at the University Hospital Hamburg-Eppendorf and REHAPORTAL, aimed to evaluate the suitability of PROMs as short-term outcome instruments within a benchmarking framework in a sample of rehabilitation facilities in routine clinical practice prior to large-scale adoption, specifically by (1) assessing the discriminatory power of disease-specific and generic PROMs to detect inter-facility differences in unadjusted comparisons, (2) identifying case-mix confounders as a basis for subsequent risk-adjusted benchmarking, (3) quantifying facility-specific performance through a pooled regression-based benchmarking approach, comparing observed outcomes to risk-adjusted expected values to ensure a transparent representation of quality differences, and by (4) developing and testing a composite outcome measure to facilitate user-friendly public reporting for patients and healthcare providers.

## Methods

### Data collection

The cooperation partners jointly developed the study protocol and PROM materials. REHAPORTAL invited a subset of its affiliated rehabilitation facilities to participate in this pilot study and thus served as the organizational framework for recruiting study sites. The entire data collection process was specifically adapted to routine clinical workflows, as these adjustments were necessary to ensure feasibility and avoid overburdening participating facilities, thereby, among other implications, limiting sample sizes per facility and precluding follow-up assessments.

Twenty-three inpatient rehabilitation facilities for total hip or total knee arthroplasty (THA, TKA) participated in this pilot study. In these facilities, only a few patients received rehabilitation treatment in day-care settings, where the therapeutic modules were identical to those of inpatient care. However, as residents, these patients were allowed to return home overnight if their health condition permitted. Consecutive samples of at least 50 patients per facility were asked to complete an online or paper-and-pencil questionnaire at admission (T0) and discharge (T1) after three weeks. Additional medical data for risk adjustment were provided by members of the care team (via online entry, the clinic information system, or paper-and-pencil forms). Inclusion criteria were a primary or secondary ICD-10 diagnosis of coxarthrosis (M16) or gonarthrosis (M17), the presence of orthopedic joint implants (Z96.6, Z98.8), and subsequent follow-up rehabilitation treatment immediately following surgery. Exclusion criteria were cognitive impairment or inability to read and understand German adequately. Due to the necessary adaptations of data collection to routine clinical workflows, information on distributed questionnaires and eligible patients was not systematically captured, preventing the calculation of response rates. Completion rates could be calculated from the submitted data. It was prespecified that in the samples of THA and TKA patients, at least *n* = 15 complete cases per facility and outcome scale should be available to be included in the benchmarking analyses of this pilot study. Data were collected from July 2018 to April 2019.

Before and after the end of the data collection period, the submitted data were repeatedly checked for completeness, validity, and plausibility. Facilities were provided with detailed reports to enable them to make corrections or supplement missing medical data, thereby increasing their respective data completeness rate or sample size for the analyses.

### Instruments

The selection of the appropriate disease-specific and generic PROMs was based on ICHOM’s recommendations [9] for THA and TKA outcome measurements, and was made in close consultation with the chief physicians of the participating rehabilitation facilities. The two disease-specific PROMs selected were supplemented by a generic PROM, which was used for both THA and TKA.

The Hip Disability and Osteoarthritis Outcome Score—Physical Function Short Form (HOOS-PS; [12, 13]) uses a set of five items on a Likert scale to evaluate hip-related difficulties in daily activities and assess physical function. The assessment uses a sum score, to which a Rasch-scaled total score is then assigned. This ranges from 0, which corresponds to extreme difficulty, to 100, which corresponds to no limitation in functional ability.

The Knee Injury and Osteoarthritis Outcome Score—Physical Function Short Form (KOOS-PS; [14]) is a seven-item Likert-scale questionnaire adapted from the KOOS [15] that measures physical function in patients with knee osteoarthritis. As with the HOOS-PS, scoring is based on a total score ranging from 0 (extreme difficulty) to 100 (no limitations), which is calculated using the Rasch method.

The VR-12 [16, 17] is a 12-item questionnaire that measures the physical and mental dimensions of health-related quality of life, and is comparable to the widely used and well-known Short-Form Health Survey 12 (SF-12). While the HOOS-PS and KOOS-PS assess disease-specific aspects, the VR-12 measures general health-related quality of life. Sum scores for physical (PCS) and mental health (MCS) are T-scores derived from the US population, with higher scores indicating better health-related quality of life.

To ensure a fair comparison between rehabilitation facilities, we collected several sociodemographic and clinical characteristics of patients known or expected to be associated with outcome levels. These variables were included in the risk adjustment.

### Data analysis

All datasets submitted by the facilities were pooled to form the total sample. To obtain the sample for the primary analyses, cases without admission and discharge scores in any PROM were excluded from further processing. All subsequent analyses were then performed separately for THA and TKA, following these basic steps: (1) calculation of unadjusted effect sizes (Cohen’s d_z_) for facilities and the pooled sample, examining the facility-level distributions of these estimates and PROM scores at admission, and assessing PROM correlations in the pooled sample; (2) risk adjustment via multiple regression, yielding effect sizes of residuals (d_res_) and ProQI (composite index of risk-adjusted PROM performance), and analyzing facility-level distributions for inter-facility benchmarking; and (3) non-completer analyses assessing potential bias in ProQI. Procedures are detailed below.

#### Objective (1)

To assess the potential of the selected PROMs to measure outcomes sensitively and to discriminate between facilities—a prerequisite for meaningful inter-facility comparisons—we examined the distribution of pre-post effect sizes d_z_ per PROM across facilities and the pooled pre-post effect size d_z_. To this end, Cohen’s d_z_ for matched samples was first calculated for each facility along with its corresponding 95% confidence interval. Additionally, Cohen’s d_z_ was calculated for the pooled sample across all patients, excluding facilities with *n* < 15 cases with complete pre-post-data for at least one PROM, as small intra-facility sample sizes were considered insufficiently representative and reliable. Cohen’s d_z_ is the mathematical equivalent of the standardized response mean (SRM), thereby accounting for correlations between pre- and post-values. The distribution of effect sizes across facilities can indicate how well the selected PROMs discriminate between facilities. For this purpose, the range and the interquartile range (IQR) of effect sizes were considered suitable indices of variability. A priori, we defined a between-facility range of at least d_z_ = 0.50 and an IQR of at least d_z_ = 0.20 as thresholds indicating sufficient discrimination. These thresholds follow the commonly used conventions for medium and small effect sizes for differences between independent samples, respectively [18]. Narrower ranges might reflect floor/ceiling effects or homogeneity, limiting statistical power for comparisons. Given the study target of at least *n* = 50 cases per facility, a priori power analysis determined that Cohen’s d_*z*_ ≥ 0.40 can be detected with 80% power (α = 0.05, two-sided paired t-tests), confirming adequate sensitivity for this study’s pre-post assessments and serving as the threshold for interpreting effect size estimates in this regard.

To evaluate individual facility performance and the discriminative potential of the PROMs, Forest plots were utilized for a visual inference approach. A facility’s pre-post improvement was considered statistically significantly different from zero at the 5% level if its 95% CI did not include zero (i.e., did not cross the vertical zero line). Likewise, we assessed inter-facility differences in unadjusted effect sizes by comparing individual 95% CIs against the pooled mean effect size. A facility was identified as showing a significantly higher or lower effect size than the average if its 95% CI did not overlap with the vertical line representing the pooled d_z_. This visual benchmarking served as an exploratory tool to identify facilities with better or worse unadjusted outcomes relative to the overall sample mean.

Furthermore, we examined the facility-level distributions of admission PROM scores to assess the potential of each PROM for discriminating between facilities. This analysis was used to determine whether baseline score variability across facilities was sufficient to enable meaningful inter-facility comparisons in general. A broad distribution of admission scores indicates not only adequate discriminatory power for unadjusted comparisons, but also underlines the importance to account for baseline case-mix variability for risk adjustment in subsequent benchmarking of facilities.

Additionally, Pearson product-moment correlations between disease-specific (HOOS-PS, KOOS-PS) and generic (VR-12 PCS, MCS) PROM scores at admission and discharge were calculated in the pooled samples to assess measurement overlap. Following proposed quality criteria for measurement properties of health status questionnaires [19], a correlation of *r* > 0.70 was considered an indicator of high convergent validity, suggesting that instruments capture nearly identical constructs or a gold standard. Consequently, we defined parallel application of PROMs as redundant if they shared more 50% of their variance (*r*^2^ > 0.50, i.e., *r* > 0.70). Conversely, PROMs were considered to capture distinct constructs when they shared less than 10% of their variance (*r*^2^ < 0.10, i.e., *r* < 0.30). On this basis, a complementary use was deemed appropriate when correlations remained below the *r* = 0.70 threshold between physical function domains, with moderate correlations (range 0.30 < *r* < 0.70) regarded as optimal for condition-specific vs. generic PROMs intended to measure related, but non-identical and thus complementary clinical constructs of health.

#### Objective (2)

Single-level multiple linear regression analyses (OLS) were performed using the pooled sample for primary analyses to predict the average outcome that could be expected at the end of rehabilitation (expected value), taking into account scores at admission to rehabilitation (baseline) on the respective outcome scales and specific patient characteristics. The selection of predictors was based on the German Pension Insurance’s (DRV) standard confounders used for single-measurement (post-only) benchmarking. The following variables were considered for risk adjustment: Age; sex; nationality (German or other); marital status (single; married; separated/divorced; widowed); educational attainment (low; medium; high); occupational status (employed; receiving a disability pension; receiving a retirement pension; not employed); whether the patient was sick or incapacitated immediately before rehabilitation; whether the rehabilitation was delivered as day-care or inpatient treatment; whether the rehabilitation was standard or expedited; the side of the surgery (left; right; or both for THA and TKA); the number of somatic diagnoses; and the presence and number of secondary psychological diagnoses. With this approach, rather than treating contextual facility characteristics as sources of bias, the analysis aimed to reflect actual performance from the patient’s perspective. Therefore, facility-specific differences, such as location, equipment, therapeutic focus, and patient-mix, represent real, decision-relevant aspects of service and treatment quality.

#### Objective (3)

To adjust for risk, patient-level confounders of age, sex, and rehabilitation baseline PROMs were consistently included in the regression models. Additional patient-level confounders were retained if statistical significance was *p* < 0.10 in a stepwise backward selection procedure. We chose the threshold of *p* < 0.10 over the more conservative level of *p* < 0.05 because we prioritized inclusivity to minimize residual confounding in observational data, thereby reducing the risk of omitting important confounders. Missing values for the confounders, HOOS-PS, and KOOS-PS were not imputed. For the VR-12, a protocol described by Buchholz and Feng [16] was applied to calculate scores for cases with missing values. The final model included all fixed variables plus those remaining after backward elimination; it was used to calculate the expected values for each patient in the facilities included in subsequent analyses. Differences between expected and observed outcomes were calculated to form patient-level residuals. These residuals were then standardized per facility by dividing the facility-specific mean residual by the facility-specific standard deviation of the differences, and their 95% CIs were computed. Although this procedure is mathematically equivalent to calculating matched-sample effect size estimates like d_z_ or SRM, allowing these standardized residuals to be expressed as effect size measures, we refer to them as effect sizes of residuals (d_res_) to distinguish them from unadjusted pre-post effect size estimates. The use of standardized residuals as effect size measures d_res_ allows for a scale-independent comparison across different PROMs. As d_res_ relies on the case-mix of the pooled sample, it represents the deviation from the case-mix-adjusted predicted outcome. Facility-specific values greater than 0 indicate a better outcome than expected based on the case-mix, while values below 0 indicate that the reported health status at discharge is below the case-mix-adjusted expectation. Similar to the unadjusted analyses, we examined the range and IQR of d_res_ per PROM across facilities, and Forest plots of d_res_ were inspected using a visual inference approach. A facility was considered to perform significantly better or worse than expected if its 95% CI did not include zero. If the entire 95% CI was located above zero, the facility’s observed outcomes significantly exceeded the outcomes predicted by the multi-variable model, whereas a 95% CI entirely below zero indicated performance significantly below the case-mix-adjusted expectation. This approach enabled a robust identification of facilities whose results were better or worse than expected while accounting for the statistical uncertainty inherent in facility-level estimates.

#### Objective (4)

Additionally, the three adjusted PROM results were combined and weighted at 50% for the specific measure, and 25% each for the two generic sum scores, to create a user-friendly composite measure for public benchmarking, the Patient-Reported Outcome Quality Index (ProQI). The ProQI was derived by converting the z-standardized residuals of all patients across facilities to a scale with a fixed mean of 90 and a standard deviation of 30, and calculating means of these values per facility. ProQI values exceeding 100 points were capped at 100, as nearly all facilities fell below this threshold per empirical distributions, ensuring interpretability while maintaining discrimination, resulting in a 0–100 scale. The weighting, scaling, and capping of the ProQI were determined normatively following discussions with experts, including representatives from the German Pension Insurance (DRV), quality managers, clinicians, and researchers. Setting the normative mean value of the ProQI at a very high level of 90 for public reporting should provide additional incentive for institutions participating in the pilot study, while a standard deviation of 30 allows for sufficient discrimination between facilities. There was also consensus that the biopsychosocial model underlying the multimodal rehabilitation approach should be reflected in the ProQI by equally weighting disease-specific and generic domains. The generic domain itself was to comprise both physical and mental components in a similar proportion. By design, the ProQI does not capture specific clinical parameters indicating the magnitude of improvements within individual facilities, but rather risk-adjusted performance differences between facilities or the extent to which a facility’s performance exceeds or falls below expected values.

#### Data completeness

For the non-completer analysis, we used the pooled sample to compare patients for whom a ProQI, relying on complete scores in all PROMs and socio-demographic as well as medical data of the patients, could be calculated (completers) with those for whom no ProQI could be calculated due to missing data (non-completers). All variables previously considered for risk adjustment, as well as PROM scores at rehabilitation admission and discharge, were included and compared using a t-test for metric variables and a chi-square test for categorical variables. In addition, we compared the ProQI of patients treated in facilities with high data completeness (rate ≥ 75%) with that of patients in facilities with low completeness (rate < 75%) using a t-test to assess potential systematic differences in ProQI related to completion rates.

After completion of the data analyses, aggregated facility-specific results were provided to the facilities in the form of individual reports. Additionally, a comprehensive overall report across all facilities was prepared. Upon request, the facilities were given access to their facility-specific data.

## Results

### Total hip arthroplasty

A total of *N* = 1,107 cases were submitted for THA (range per facility: *n* = 8 to 123; median = 49 cases) by K = 23 facilities. After excluding cases with missing scores in all PROMs at both time points (T0 and T1), *n* = 961 cases remained eligible for primary analyses requiring complete longitudinal data in at least one PROM (range per facility: *n* = 1 to 88; median = 46 cases). This corresponds to an initial data completeness rate of 86%. Information on the socio-demographic and clinical characteristics of the sample is provided in Table 1.

**Table 1:** Characteristics of THA patients across all facilities at admission (*n* = 961)

Variable	
Mean Age (SD)	67.32 (11.05)
Sex (Female)	56.2%
Education (High school degree)	18.8%
Nationality (only German)	98.7%
Marital status (married)	63.0%
Occupational status (full or part-time employed)	27.2%
Receiving a disability pension	2.8%
Setting of rehabilitation (inpatient)	98.6%
Standard rehabilitation in an expedited procedure	6.5%
Work incapacity at admission	22.4%
More than five somatic diagnoses	18.4%
One or more mental disorders	5.8%
Side of surgery (both sides)	3.8%
Median time (in days) since surgery (IQR)	11 (8–15)
THA-related diagnosis (ICD-10)	
M16	45.2%
M16 & Z96.64	30.1%
Z96.64	5.9%
M16 & Z98.8	3.4%
Z96.64 & S72.01	0.9%
T84.04	0.6%
T84.04 & Z96.64	0.6%
other	13.3%

#### Objective (1)

The upper section of Table 2 presents comprehensive within-facility descriptive statistics for all facilities (including those with *n* < 15 cases), while the lower section provides between-facility distributional summaries and pooled results excluding facilities with *n* < 15 cases per prespecified criteria (see caption for details). After these exclusions, facility-specific data completeness rates were 32–100% for HOOS-PS (median = 79%; IQR: 72–90%; k = 20), 61–100% for VR-12 PCS (median = 92%; IQR: 87–96%; k = 21), and 61–100% for VR-12 MCS (median = 91%; IQR: 85–94%; k = 21).

**Table 2:** THA: Facility-specific unadjusted descriptive results and distributional summaries across facilities for HOOS-PS, VR-12 PCS, and VR-12 MCS at admission (T0) and discharge (T1)

	**All**	**HOOS-PS**								**VR-12 PCS**								**VR-12 MCS**							
**Facility**	***N***	***n***	**M t0**	**SD t0**	**M t1**	**SD t1**	**d**_**z**_	***p***	**r**_**t0-t1**_	***n***	**M t0**	**SD t0**	**M t1**	**SD t1**	**d**_**z**_	***p***	**r**_**t0-t1**_	***n***	**M t0**	**SD t0**	**M t1**	**SD t1**	**d**_**z**_	***p***	**r**_**t0-t1**_
A	32	25	43.96	21.11	53.27	18.22	0.40	0.058	0.30	29	27.07	9.10	30.47	7.53	0.46	0.019	0.62	29	50.94	13.61	51.37	13.83	0.05	0.793	0.80
B	74	43	54.40	19.47	70.33	16.03	0.76	< 0.001	0.32	45	26.83	9.30	36.28	8.57	0.92	< 0.001	0.35	45	47.07	12.01	53.88	11.80	0.53	< 0.001	0.43
C	9	5	32.74	10.95	66.60	14.26	1.40	0.035	− 0.84	6	25.83	7.46	35.18	10.49	1.11	0.042	0.61	6	41.44	15.31	57.26	11.42	0.83	0.097	0.01
D	57	18	55.63	24.57	75.11	17.65	1.23	< 0.001	0.76	50	30.16	9.72	38.68	7.44	0.91	< 0.001	0.43	49	48.94	12.67	56.70	9.94	0.67	< 0.001	0.49
E	18	13	37.33	20.11	55.22	15.15	1.11	0.002	0.61	17	25.95	5.42	34.03	9.01	0.92	0.002	0.34	17	42.73	14.90	48.09	11.76	0.47	0.071	0.66
F	34	28	49.66	18.48	54.39	17.30	0.33	0.088	0.69	32	27.74	9.23	34.65	8.00	0.80	< 0.001	0.50	32	49.57	12.80	51.98	12.15	0.25	0.173	0.69
G	56	56	43.55	20.86	62.04	15.92	1.03	< 0.001	0.56	56	29.80	8.75	35.16	9.23	0.54	< 0.001	0.38	56	47.15	12.93	54.76	10.99	0.68	< 0.001	0.57
H	49	44	45.54	24.41	63.83	17.97	0.72	< 0.001	0.32	46	28.32	8.84	36.17	7.38	0.84	< 0.001	0.34	46	43.87	13.39	50.92	10.82	0.60	< 0.001	0.55
I	57	49	46.82	17.62	64.73	15.64	1.06	< 0.001	0.49	49	25.57	7.62	34.73	7.14	1.04	< 0.001	0.28	49	47.03	12.03	53.40	8.91	0.68	< 0.001	0.63
J	8	1	44.10	n.a	91.20	n.a	n.a	n.a	n.a	1	23.66	n.a	52.41	n.a	n.a	n.a	n.a	1	25.77	n.a	49.69	n.a	n.a	n.a	n.a
K	28	27	44.46	20.34	56.81	20.09	0.55	0.008	0.38	27	25.73	8.86	31.71	8.91	0.71	0.001	0.54	27	49.15	13.72	51.86	15.74	0.20	0.315	0.57
L	44	35	48.18	21.89	57.42	18.68	0.45	0.012	0.50	40	29.37	9.37	34.51	8.40	0.58	< 0.001	0.50	40	46.92	14.57	51.16	13.07	0.39	0.018	0.69
M	59	45	45.80	16.24	57.01	19.06	0.49	0.002	0.17	54	28.79	7.86	34.46	7.60	0.69	< 0.001	0.44	54	48.85	13.63	55.57	10.89	0.49	< 0.001	0.39
N	33	24	46.72	24.91	63.34	16.02	0.80	< 0.001	0.55	30	32.47	11.57	39.47	8.71	0.71	< 0.001	0.56	29	45.19	14.78	51.71	13.66	0.54	0.007	0.64
O	54	36	48.06	20.34	65.13	16.32	0.63	< 0.001	− 0.08	52	31.21	9.28	35.06	8.95	0.31	0.029	0.08	49	46.83	13.41	55.50	10.29	0.56	< 0.001	0.15
P	48	48	51.68	20.54	61.74	17.02	0.44	0.004	0.26	48	27.06	9.56	34.23	7.95	0.69	< 0.001	0.30	48	50.18	12.22	52.69	11.52	0.23	0.118	0.58
Q	60	54	35.31	23.26	55.81	19.62	0.82	< 0.001	0.33	58	27.40	7.60	33.04	8.30	0.63	< 0.001	0.36	58	40.96	14.14	49.58	13.35	0.70	< 0.001	0.60
R	48	38	42.92	19.00	67.95	17.68	1.12	< 0.001	0.26	44	27.39	7.79	38.15	9.07	0.95	< 0.001	0.10	41	47.35	14.13	53.30	11.19	0.33	0.042	−0.01
S	31	26	45.82	20.99	57.99	16.20	0.60	0.005	0.43	27	28.66	7.25	34.52	7.03	0.67	0.002	0.25	26	44.42	11.91	50.92	10.03	0.67	0.002	0.62
T	61	44	44.33	20.58	58.06	16.49	0.66	< 0.001	0.38	52	27.73	8.44	35.55	8.12	0.72	< 0.001	0.14	51	46.32	14.11	50.19	14.43	0.36	0.014	0.71
U	73	45	44.42	19.96	61.33	15.37	0.88	< 0.001	0.44	59	28.37	8.60	34.99	8.51	0.71	< 0.001	0.41	56	47.08	12.14	51.21	13.04	0.32	0.019	0.49
V	51	51	36.08	17.33	74.60	17.49	1.80	< 0.001	0.25	51	22.65	8.05	39.26	7.53	1.44	< 0.001	− 0.09	51	58.06	13.49	62.48	6.60	0.34	0.021	0.29
W	123	88	44.15	20.61	61.44	14.67	0.85	< 0.001	0.38	88	26.45	8.42	37.21	8.69	1.05	< 0.001	0.29	87	48.18	14.73	53.41	12.44	0.36	0.001	0.42
**Facility level**	**k = 23**	**k = 20**								**k = 21**								**k = 21**							
Min	8.0	18.00	35.31	16.24	53.27	14.67	0.33		− 0.08	17.00	22.65	5.42	30.47	7.03	0.31		− 0.09	17.00	40.96	11.91	48.09	6.60	0.05		−0.01
PCT 25	32.5	27.75	44.10	19.35	57.32	16.03	0.54		0.29	32.00	26.83	7.86	34.46	7.53	0.67		0.28	32.00	46.32	12.67	51.16	10.82	0.33		0.43
Median	49.0	43.50	45.67	20.56	61.59	17.16	0.74		0.38	48.00	27.73	8.75	34.99	8.30	0.71		0.35	48.00	47.08	13.49	51.98	11.76	0.47		0.57
PCT 75	58.0	48.25	48.09	21.31	64.83	18.03	0.92		0.49	52.00	28.79	9.28	36.28	8.71	0.92		0.44	51.00	48.94	14.13	53.88	13.07	0.60		0.64
Max	123.0	88.00	55.63	24.91	75.11	20.09	1.80		0.76	88.00	32.47	11.57	39.47	9.23	1.44		0.62	87.00	58.06	14.90	62.48	15.74	0.70		0.80
Range	115.0	70.00	20.32	8.67	21.84	5.42	1.47		0.84	71.00	9.82	6.15	9.00	2.20	1.13		0.71	70.00	17.10	2.99	14.39	9.14	0.65		0.81
IQR	25.5	20.50	3.99	1.95	7.51	2.01	0.38		0.20	20.00	1.96	1.42	1.82	1.18	0.25		0.16	19.00	2.62	1.46	2.72	2.25	0.27		0.21
**Pooled data**	1,107	824	45.21	20.85	62.20	17.75	0.76	< 0.001	0.35	954	27.82	8.83	35.57	8.41	0.76	< 0.001	0.31	940	47.60	13.71	53.23	12.04	0.45	< 0.001	0.53

Upper section: Facility-specific results (*n*, mean T0/T1 scores, SDs, unadjusted paired-sample effect sizes (Cohen's d_z_), paired-sample *t*-test *p*-values, pre/post correlations), including facilities with *n* < 15 cases. Lower section: Distributional summaries across facilities (k: number of facilities with *n* ≥ 15, min, max, median, quartiles, range, IQR) and pooled results, excluding facilities with *n* < 15 per prespecified criteria

The HOOS-PS score at admission correlated with the Physical Component Summary (PCS) of the VR-12 and the Mental Component Summary (MCS) of the VR-12, with correlations of *r* = 0.48 (*p* < 0.001) and *r* = 0.22 (*p* < 0.001), respectively. The two VR-12 scales correlated with *r* = − 0.08 (*p* = 0.019). Correlations at discharge were *r* = 0.56 (*p* < 0.001), *r* = 0.34 (*p* < 0.001), and *r* = 0.19 (*p* < 0.001), respectively.

**Fig. 1:**
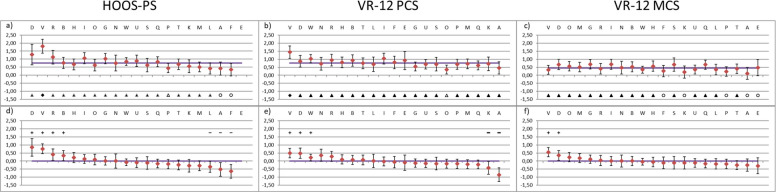
Forest plots using the visual inference approach for THA patients in 21 rehabilitation facilities: (panels a-c) unadjusted pre-post effect sizes d_z_ and (panels d-f) effect sizes of residuals d_res_ after risk adjustment (all panels sorted by panels d–f: statistical significance first, then effect size magnitude), with 95% confidence intervals for HOOS-PS, VR-12 PCS, and VR-12 MCS across rehabilitation facilities (*n* ≥ 15 cases). Facilities C, E, and J (HOOS-PS) and C, J (VR-12 PCS/MCS) were excluded due to *n* < 15. Facility E (*n* < 15 for HOOS-PS) is represented with empty data points in panel (a) for HOOS-PS, while included for VR-12 PROMs, to maintain visual consistency across all forest plots. Panels **a-c**: Effect sizes d_z_ indicate observed improvement in patients (higher = greater). CIs including zero indicate non-significant pre-post change; CIs not overlapping the line of the pooled mean effect size d_z_ (purple) indicate facilities above/below-average observed performance. Symbols: ○ non-significant pre-post; △ significant pre-post, below pooled d_z_; ▲ significant, at pooled d_z_; ◆ significant, above pooled d_z_. Panels **d-f**: Effect sizes of residuals d_res_ after risk adjustment (higher = greater improvement than case-mix expected). CIs including zero indicate performance not significantly different from case-mix expectation; CIs excluding zero indicate significantly better/worse expected performance. Symbols: + significantly better; - significantly worse than expected

The mean HOOS-PS scores at admission per facility ranged from 35.31 to 55.63 (IQR: 44.10–48.09; Table 2). Effect sizes for HOOS-PS changes from admission to discharge (Cohen’s d_z_) had a range of d_z_ = 1.47 (0.33 to 1.80) across facilities, and an IQR of d_z_ = 0.38 (0.54 to 0.92). The pooled sample effect size across all patients was d_z_ = 0.76 (95% CI, 0.69–0.84). Panel (a) of Fig. 1 presents unadjusted effect sizes with 95% confidence intervals (CI) for facilities with *n* ≥ 15 cases and a reference line indicating the pooled average effect size across all patients. Facilities A and F showed effect sizes not significantly different from zero (per CIs), facility V was significantly above average, and facility P was significantly below average.

Admission scores for the Physical Component Summary (PCS) of the VR-12 across facilities ranged from 22.65 to 32.47 (IQR: 26.83–28.79; Table 2). Facility-level unadjusted effect sizes (d_z_) for VR-12 PCS changes had a range of d_z_ = 1.13 (0.31 to 1.44), and an IQR of d_z_ = 0.25 (0.67 to 0.92). The pooled effect size across all patients was d_z_ = 0.76 (95% CI, 0.69–0.84). Panel (b) of Fig. 1 displays unadjusted effect sizes and 95% CIs for facilities with *n* ≥ 15 cases, with a reference line showing the overall pooled estimate. Per CIs, all facilities exhibited effect sizes significantly different from zero; facility V significantly exceeded the average, and facility O fell significantly below.

Across facilities, mean admission scores for the Mental Component Summary (MCS) of the VR-12 spanned 40.96 to 58.06 (IQR: 46.32–48.94; Table 2). Facility-specific unadjusted effect sizes (d_z_) for VR-12 MCS changes from admission to discharge had a range of d_z_ = 0.65 (0.05 to 0.70), and an IQR of d_z_ = 0.27 (0.33 to 0.60). Pooled across all patients, the effect size was d_z_ = 0.45 (95% CI, 0.38–0.51). Panel (c) of Fig. 1 illustrates unadjusted effect sizes with 95% CIs for facilities meeting *n* ≥ 15 criteria, including a reference line for the overall pooled effect size. Based on CIs, for five facilities (F, K, P, A, E), the observed effect sizes are not statistically significantly different from zero. No facility significantly exceeded the pooled average or fell significantly below it.

#### Objective (2)

Table 3 reports beta weights, coefficient p-values, and corrected R^2^ from multiple linear regression models predicting discharge HOOS-PS, VR-12 PCS, and VR-12 MCS as primary analyses for risk-adjusted benchmarking in the THA sample.

**Table 3:** Confounders included in the three risk adjustment models to predict discharge scores (THA, *n* = 961)

Confounder	HOOS-PS		VR-12 PCS		VR-12 MCS	
	**beta**	***p***	**beta**	***p***	**beta**	***p***
HOOS-PS at admission	0.301	< 0.001	-	-	-	-
VR-12 PCS at admission	-	-	0.310	< 0.001	-	-
VR-12 MCS at admission	0.137	< 0.001	0.213	< 0.001	0.515	< 0.001
Female	− 0.121	0.001	-	-	− 0.046	
High school degree	0.089	0.004	0.061	0.039	0.063	0.028
Work incapacity at admission	− 0.097	0.020	-	-	− 0.082	0.009
Number of somatic diagnoses	-	-	-	-	− 0.073	0.002
More than five somatic diagnoses	-	-	− 0.084	0.024	-	-
Adjusted R^2^	0.165		0.144		0.289	

Confounders to predict discharge scores of HOOS-PS, VR-12 PCS, and MCS with beta weights, coefficient p-values, and adjusted R^2^ for the respective model

“-“ = confounder not significant (*p* > 0.10) and not included in the final model for the respective outcome. Tested confounders not significant in any of the three models are omitted from the table

#### Objective (3)

Based on the regression models, the effect sizes of residuals (d_res_) for HOOS-PS had range = 1.49 (− 0.64 to 0.85) and IQR = 0.41 (− 0.25 to 0.15); for VR-12 PCS range = 1.34 (− 0.85 to 0.49) and IQR = 0.26 (− 0.16 to 0.10); and for VR-12 MCS range = 0.85 (− 0.30 to 0.55) and IQR = 0.20 (− 0.16 to 0.04; Table 4). Panels (d–f) of Fig. 1 display facility-specific d_res_ with 95% CIs for facilities with *n* ≥ 15 (x-axis/zero line indicates no deviation from expected outcomes). Per CIs, significant deviations from zero occurred for: HOOS-PS (D, V, R, B above; L, A, F below); VR-12 PCS (V, D, W above; K, A below); VR-12 MCS (V, D above).

**Table 4:** Completion rates and effect sizes of residuals (d_res_) for HOOS-PS, VR-12 PCS, MCS, and ProQI in THA

	**All**	**HOOS-PS**			**VR-12 PCS**			**VR-12 MCS**			**THA ProQI**		
**Facility**	***N***	***n***	**CR (%)**	**d**_**res**_	***n***	**CR (%)**	**d**_**res**_	***n***	**CR (%)**	**d**_**res**_	***n***	**CR (%)**	**ProQI**
A	32	24	75	− 0.52	29	91	− 0.85	28	88	− 0.25	24	75	75.4
B	74	43	58	0.34	45	61	0.07	45	61	0.00	43	58	95.3
C	9	5	56		6	67		6	67		5	56	
D	57	17	30	0.85	41	72	0.48	46	81	0.35	16	28	100.0
E	18	13	72		17	94	− 0.10	17	94	− 0.30	13	72	
F	34	26	76	− 0.64	32	94	− 0.07	30	88	− 0.11	26	76	80.3
G	56	56	100	0.01	56	100	− 0.13	56	100	0.12	56	100	89.8
H	49	40	82	0.22	45	92	0.10	42	86	− 0.06	39	80	93.7
I	57	49	86	0.13	49	86	− 0.03	49	86	0.00	49	86	91.4
J	8	1	13		1	13		1	13		1	13	
K	28	27	96	− 0.29	27	96	− 0.42	27	96	− 0.12	27	96	81.2
L	44	33	75	− 0.36	23	52	0.01	38	86	− 0.18	17	39	83.9
M	59	44	75	− 0.29	49	83	− 0.20	50	85	0.18	39	66	85.0
N	33	20	61	0.00	29	88	0.36	26	79	0.00	20	61	92.3
O	54	35	65	0.10	50	93	− 0.16	49	91	0.24	35	65	92.3
P	48	48	100	− 0.19	48	100	− 0.17	48	100	− 0.18	48	100	84.8
Q	60	52	87	− 0.16	56	93	− 0.21	55	92	− 0.16	50	83	84.3
R	48	33	69	0.40	41	85	0.28	38	79	0.04	33	69	99.2
S	31	25	81	− 0.12	26	84	− 0.16	26	84	− 0.11	25	81	86.8
T	61	43	70	− 0.24	41	67	0.07	50	82	− 0.25	36	59	85.5
U	73	42	58	− 0.10	57	78	− 0.15	52	71	− 0.12	42	58	86.6
V	51	51	100	0.76	51	100	0.49	51	100	0.55	51	100	100.0
W	123	88	72	− 0.08	85	69	0.22	84	68	− 0.04	84	68	90.4
**Facility level**	**K = 23**	**k = 20**			**k = 21**			**k = 21**			**k = 20**		
Min	8	17	30	− 0.64	17	52	− 0.85	17	61	− 0.30	16	28	75.4
PCT 25	32.5	27	68	− 0.25	29	78	− 0.16	30	81	− 0.16	26	60	84.7
Median	49	41	75	− 0.09	45	88	− 0.07	46	86	− 0.06	38	72	88.3
PCT 75	58	48	86	0.15	50	94	0.10	50	92	0.04	48	84	92.7
Max	123	88	100	0.85	85	100	0.49	84	100	0.55	84	100	100.0
Range	115	71	70	1.49	68	48	1.34	67	39	0.85	68	72	24.6
IQR	25.5	22	18	0.41	21	16	0.26	20	11	0.20	23	24	8.0

Completion Rates: For the HOOS-PS, VR-12 PCS, and MCS, rates refer to cases with complete PROM scores and all required risk-adjustment variables (socio-demographic/medical) at both admission and discharge; the ProQI completion rate further requires full data availability across all three PROM scales simultaneously. d_res_ (Effect Size of Residuals): A risk-adjusted, scale-independent measure representing the difference between a patient’s observed discharge score and the value predicted by the regression model, expressed as a standardized effect size. ProQI (Patient-Reported Outcome Quality Index): A composite index (0–100) aggregating weighted, risk-adjusted residuals across disease-specific and generic health domains to benchmark facility performance. Upper section: Facility-specific results (n, completion rates (submitted data/sufficiently complete data), effect sizes of residuals (d_res_), composite Patient-reported Quality Index (ProQI)), including facilities with *n* < 15 cases. Lower section: Distributional summaries across facilities (k: number of facilities with *n* ≥ 15, min, max, median, quartiles, range, IQR), excluding facilities with *n* < 15 per prespecified criteria

#### Objective (4)

ProQI composite for THA had a range = 24.6 (75.4 to 100) and IQR = 8.0 (84.7 to 92.7; Table 4; Supplementary Fig. S1), corresponding to Cohen’s d of 0.82 (range) and 0.27 (IQR) on the ProQI scale (M = 90, SD = 30). Due to insufficiently complete data, no ProQI could be calculated for facilities C, E, and J.

#### Data completeness

Post-regression completeness rates were 30–100% for HOOS-PS (median = 75%; IQR: 68–86%; k = 20), 52–100% for VR-12 PCS (median = 88%; IQR: 78–94%; k = 21), and 61–100% for VR-12 MCS (median = 86%; IQR: 81–92%; k = 21; Table 4). Across all facilities, *n* = 760 cases yielded complete ProQI data, corresponding to an overall completion rate of 69% (median = 72%; range: 28–100%, IQR: 60–84%).

Further non-completer analyses showed that non-completers (cases for which no patient-level ProQI could be computed) had higher baseline VR-12 PCS values by 2.04 points (*t* = − 3.305; df = 1048; *p* < 0.01; Supplementary Table S1). No other tested differences reached statistical significance (Supplementary Tables S2 & S3). With regard to the ProQI, there were no significant differences between patients in facilities with high completion rates and those in facilities with low completion rates (mean difference = − 1.73; *t* = − 1.050; df = 758; *p* = 0.294; Supplementary Table S4).

### Total knee arthroplasty

A total of *N* = 1,139 TKA cases were submitted by K = 23 facilities (facility range: *n* = 10 to 126; median = 45 cases). After excluding cases with missing PROM scores at both time points (T0/T1), *n* = 961 remained eligible for primary analyses requiring complete longitudinal data in at least one PROM (facility range: *n* = 2 to 85; median = 41 cases), yielding 84% initial data completeness. Socio-demographic and clinical characteristics are shown in Table 5.

**Table 5:** Characteristics of TKA patients across all facilities at admission (*n* = 961)

Variable	
Mean Age (SD)	67.06 (9.91)
Sex (Female)	61.0%
Education (High school degree)	14.7%
Nationality (only German)	96.1%
Marital status (married)	68.9%
Occupational status (full or part-time employed)	27.8%
Receiving a disability pension	3.8%
Setting of rehabilitation (inpatient)	98.3%
Standard rehabilitation in an expedited procedure	6.9%
Work incapacity at admission	24.2%
More than five somatic diagnoses	22.4%
One or more mental disorders	5.7%
Site of surgery (both sides)	2.9%
Median time (in days) since surgery (IQR)	11 (8–15)
TKA-related diagnosis (ICD-10)	
M17	50.2%
M17 + Z96.65	32.9%
Z96.65	4.1%
M17 + Z98.8	3.2%
T84.05 + Z96.65	1.5%
T84.05	0.8%
Z98.8	0.5%
other	6.8%

#### Objective (1)

The upper section of Table 6 presents comprehensive within-facility descriptive statistics for all facilities (including those with *n* < 15 cases), while the lower section provides between-facility distributional summaries and pooled results excluding facilities with *n* < 15 cases per prespecified criteria (see caption for details). After exclusions, data completeness rates were 59–100% for KOOS-PS (median = 91%; IQR: 79–97%; k = 21), 63–100% for VR-12 PCS (median = 95%; IQR: 79–98%; k = 21), and 63–100% for VR-12 MCS (median = 95%; IQR: 79–98%; k = 21).

**Table 6:** TKA: Facility-specific unadjusted descriptive results and distributional summaries across facilities for KOOS-PS, VR-12 PCS, and VR-12 MCS at admission (T0) and discharge (T1)

	**All**	**KOOS-PS**								**VR-12 PCS**								**VR-12 MCS**							
**Facility**	**N**	***n***	**M t0**	**SD t0**	**M t1**	**SD t1**	**d**_**z**_	***p***	**r**_**t0-t1**_	***n***	**M t0**	**SD t0**	**M t1**	**SD t1**	**d**_**z**_	***p***	**r**_**t0-t1**_	***n***	**M t0**	**SD t0**	**M t1**	**SD t1**	**d**_**z**_	***p***	**r**_**t0-t1**_
A	49	47	51.09	17.15	58.89	17.32	0.52	< 0.001	0.62	47	27.94	7.82	31.61	8.10	0.43	0.005	0.42	47	48.00	15.12	52.02	13.60	0.34	0.023	0.67
B	59	35	54.26	12.93	63.59	8.24	0.87	< 0.001	0.57	37	27.93	7.08	35.38	6.31	0.95	< 0.001	0.32	37	43.72	13.93	48.84	12.11	0.53	0.003	0.74
C	30	8	50.34	22.35	61.50	11.43	0.65	0.111	0.65	8	30.67	7.48	36.40	8.46	0.78	0.063	0.58	8	46.51	16.35	50.37	12.20	0.72	0.080	0.97
D	62	47	54.20	13.99	70.54	12.36	1.12	< 0.001	0.39	49	29.75	8.05	37.54	7.31	1.02	< 0.001	0.51	47	50.00	12.70	53.54	12.06	0.31	0.041	0.57
E	34	32	49.68	13.13	64.15	15.71	1.22	< 0.001	0.67	32	29.44	9.50	35.40	9.97	0.40	0.032	− 0.20	32	49.85	14.03	51.46	12.74	0.11	0.549	0.38
F	34	33	52.51	13.45	59.78	14.01	0.54	0.004	0.51	34	31.45	7.65	35.20	8.16	0.49	0.007	0.53	34	43.88	13.31	49.66	10.13	0.55	0.003	0.63
G	39	38	50.97	15.64	59.47	14.56	0.53	0.002	0.44	39	27.82	8.34	34.45	7.84	0.76	< 0.001	0.41	39	45.30	14.37	50.64	11.81	0.43	0.011	0.56
H	52	51	43.45	20.27	62.31	14.37	1.22	< 0.001	0.65	51	29.62	8.55	36.70	7.73	0.90	< 0.001	0.54	51	44.77	11.97	49.78	11.34	0.51	0.001	0.64
I	53	42	47.44	17.74	61.51	11.87	1.15	< 0.001	0.73	42	26.11	7.49	32.32	7.78	0.69	< 0.001	0.31	42	44.37	16.20	50.58	13.29	0.60	< 0.001	0.77
J	10	1	42.10	n.a	58.00	n.a	n.a	n.a	n.a	2	39.15	11.06	44.05	10.13	n.a	n.a	n.a	2	66.99	5.13	48.63	21.04	n.a	n.a	n.a
K	38	33	52.93	15.34	60.42	10.68	0.56	0.003	0.53	33	30.04	8.55	32.32	8.82	0.30	0.098	0.61	33	45.71	14.14	48.83	13.27	0.32	0.079	0.74
L	45	38	49.43	14.51	62.29	12.76	0.77	< 0.001	0.26	38	27.01	8.32	35.78	7.70	1.01	< 0.001	0.42	38	45.65	13.97	52.58	9.70	0.57	0.001	0.52
M	69	51	49.91	15.15	59.36	12.02	0.66	< 0.001	0.46	51	28.77	8.54	32.43	8.20	0.40	0.006	0.40	50	49.15	14.55	52.83	12.77	0.41	0.006	0.79
N	40	38	46.71	17.24	54.36	15.54	0.61	< 0.001	0.71	38	28.59	7.62	31.51	7.58	0.47	0.007	0.66	38	41.78	14.38	44.64	14.77	0.33	0.051	0.82
O	43	40	49.61	14.05	63.02	13.19	1.12	< 0.001	0.62	41	26.63	7.83	34.70	7.75	0.91	< 0.001	0.35	41	46.74	14.09	50.27	12.56	0.29	0.066	0.60
P	35	34	49.07	16.22	62.86	8.57	1.09	< 0.001	0.63	35	28.75	7.95	34.43	6.47	0.61	0.001	0.17	35	47.15	11.79	52.61	11.10	0.43	0.015	0.39
Q	75	68	43.63	21.15	56.97	12.92	0.62	< 0.001	0.27	73	27.69	9.15	33.66	9.00	0.61	< 0.001	0.41	73	41.58	13.73	47.47	12.75	0.46	< 0.001	0.54
R	43	39	48.45	16.20	63.98	10.19	0.86	< 0.001	0.12	42	30.56	7.87	34.13	7.52	0.37	0.023	0.20	42	43.48	14.17	49.22	10.18	0.51	0.002	0.61
S	24	24	44.96	12.95	58.70	13.87	1.07	< 0.001	0.54	24	25.02	7.23	33.87	6.91	1.29	< 0.001	0.53	23	43.69	13.55	49.68	14.17	0.74	0.002	0.83
T	53	48	51.29	15.23	62.64	11.85	0.92	< 0.001	0.61	49	28.50	7.83	35.85	7.66	0.95	< 0.001	0.50	49	46.80	11.35	49.91	12.30	0.26	0.070	0.51
U	65	48	48.30	18.27	60.04	13.26	0.77	< 0.001	0.57	49	26.97	7.92	33.20	7.83	0.71	< 0.001	0.39	49	46.13	13.42	50.34	12.74	0.35	0.018	0.57
V	61	61	33.70	22.83	76.34	11.91	1.81	< 0.001	0.20	61	22.34	8.67	39.99	6.79	1.48	< 0.001	− 0.17	61	57.93	12.65	61.76	9.40	0.30	0.022	0.36
W	126	85	44.25	17.72	62.38	9.48	1.07	< 0.001	0.35	85	26.94	7.89	35.44	8.01	0.94	< 0.001	0.35	85	46.45	13.67	50.59	12.21	0.31	0.005	0.47
**Facility level**	**k = 23**	**k = 21**								**k = 21**								**k = 21**							
Min	10.0	24.00	33.70	12.93	54.36	8.24	0.52		0.12	24.00	22.34	7.08	31.51	6.31	0.30		− 0.20	23.00	41.58	11.35	44.64	9.40	0.11		0.36
PCT 25	36.5	35.00	46.71	14.05	59.47	11.85	0.62		0.39	37.00	26.97	7.82	33.20	7.52	0.47		0.32	37.00	43.88	13.31	49.66	11.34	0.31		0.52
Median	45.0	40.00	49.43	15.64	62.29	12.76	0.87		0.54	42.00	27.94	7.92	34.45	7.75	0.71		0.41	42.00	45.71	13.93	50.34	12.30	0.41		0.60
PCT 75	60.0	48.00	51.09	17.72	63.02	14.01	1.12		0.62	49.00	29.44	8.54	35.44	8.10	0.95		0.51	49.00	47.15	14.17	52.02	12.77	0.51		0.74
Max	126.0	85.00	54.26	22.83	76.34	17.32	1.81		0.73	85.00	31.45	9.50	39.99	9.97	1.48		0.66	85.00	57.93	16.20	61.76	14.77	0.74		0.83
Range	116.0	61.00	20.56	9.90	21.98	9.08	1.29		0.61	61.00	9.11	2.42	8.48	3.66	1.18		0.86	62.00	16.35	4.85	17.12	5.37	0.63		0.47
IQR	23.5	13.00	4.38	3.67	3.55	2.16	0.50		0.23	12.00	2.47	0.72	2.24	0.58	0.48		0.19	12.00	3.27	0.86	2.36	1.43	0.20		0.22
**Pooled data**	1,139	932	47.69	17.55	62.33	13.46	0.83	< 0.001	0.38	950	27.84	8.32	34.72	8.05	0.71	< 0.001	0.31	946	46.49	14.05	51.00	12.50	0.39	< 0.001	0.63

Upper section: Facility-specific results (n, mean T0/T1 scores, SDs, unadjusted paired-sample effect sizes (Cohen’s d_z_), paired-sample t-test p-values, pre/post correlations), including facilities with *n* < 15 cases. Lower section: Distributional summaries across facilities (k: number of facilities with *n* ≥ 15, min, max, median, quartiles, range, IQR) and pooled results, excluding facilities with *n* < 15 per prespecified criteria

KOOS-PS at admission correlated with the Physical Component Summary (PCS) of the VR-12 with *r* = 0.50 (*p* < 0.001), and with the Mental Component Summary (MCS) of the VR-12 with *r* = 0.24 (*p* < 0.001). The two VR-12 scales correlated with *r* = − 0.11 (*p* < 0.001). Correlations at discharge were *r* = 0.56 (*p* < 0.001; KOOS-PS/PCS), *r* = 0.43 (*p* < 0.001; KOOS-PS/MCS), and *r* = 0.18 (*p* < 0.001; PCS/MCS), respectively.

**Fig. 2:**
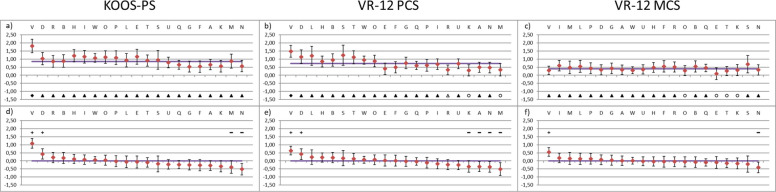
Forest plots using the visual inference approach for TKA patients in 21 rehabilitation facilities: (panels a–c) unadjusted pre-post effect sizes d_z_ and (panels d–f) effect sizes of residuals d_res_ after risk adjustment (all panels sorted by panels d–f: statistical significance first, then effect size magnitude), with 95% confidence intervals for KOOS-PS, VR-12 PCS, and VR-12 MCS across rehabilitation facilities (n ≥ 15 cases). Facilities C and J were excluded due to n < 15. Panels **a**–**c**: Effect sizes d_z_ indicate observed improvement in patients (higher = greater). CIs including zero indicate non-significant pre-post change; CIs not overlapping the line of the pooled mean effect size d_z_ (purple) indicate facilities above/below-average observed performance. Symbols: ○ non-significant pre-post; △ significant pre-post, below pooled d_z_; ▲ significant, at pooled d_z_; ◆ significant, above pooled d_z_. Panels **d**–**f**: Effect sizes of residuals d_res_ after risk adjustment (higher = greater improvement than case-mix expected). CIs including zero indicate performance not significantly different from case-mix expectation; CIs excluding zero indicate significantly better/worse expected performance. Symbols: + significantly better; - significantly worse than expected

Facility-specific mean admission KOOS-PS scores spanned 33.70 to 54.26 (IQR: 46.71–51.09; Table 6). KOOS-PS change effect sizes (Cohen’s d_z_) across facilities had a range of d_z_ = 1.29 (0.52 to 1.81), and an IQR of d_z_ = 0.50 (0.62 to 1.12). Pooled effect size across all patients was d_z_ = 0.83 (95% CI, 0.75–0.90). Panel (a) of Fig. 2 shows unadjusted effect sizes for facilities with *n* ≥ 15 cases, and the reference line for the pooled average. According to CIs, all facilities showed significant effect sizes, with facility V significantly exceeding the average.

VR-12 PCS admission scores per facility varied from 22.34 to 31.45 (IQR: 26.97–29.44; Table 6). Unadjusted VR-12 PCS effect sizes (d_z_) had a range of d_z_ = 1.18 (0.30 to 1.48), and an IQR of d_z_ = 0.48 (0.47 to 0.95). Overall pooled effect size was d_z_ = 0.71 (95% CI, 0.64–0.79). Panel (b) of Fig. 2 depicts these effect sizes with 95% CIs for facilities with *n* ≥ 15 cases, reference line indicating pooled estimate. All facilities except K and M showed significant effect sizes (per CIs); facility V was significantly above and facility R below average.

Mean VR-12 MCS admission scores ranged 41.58–57.93 across facilities (IQR: 43.88–47.15; Table 6). Facility-level VR-12 MCS effect sizes (d_z_) had a range of d_z_ = 0.63 (0.11 to 0.74), and an IQR of d_z_ = 0.20 (0.31 to 0.51). Pooled patient-level effect size was d_z_ = 0.39 (95% CI, 0.32–0.47). Panel (c) Fig. 2 presents unadjusted effect sizes and 95% CIs for facilities with *n* ≥ 15 cases with a pooled effect size reference line. Four facilities (O, E, T, K) showed non-significant effect sizes (per CIs); none significantly deviated from the pooled average effect size.

#### Objective (2)

Table 7 reports beta weights, coefficient p-values, and corrected R^2^ from multiple linear regression models predicting discharge KOOS-PS, VR-12 PCS, and VR-12 MCS as primary analyses for risk-adjusted benchmarking in the TKA sample.

**Table 7:** Confounders included in the three risk adjustment models to predict discharge scores (TKA, *n* = 961)

Confounder	KOOS-PS		VR-12 PCS		VR-12 MCS	
	**beta**	***p***	**beta**	***p***	**beta**	***p***
KOOS-PS at admission	0.228	< 0.001	-	-	-	-
VR-12 PCS at admission	0.134	< 0.001	0.328	< 0.001	0.075	0.009
VR-12 MCS at admission	0.325	< 0.001	0.247	< 0.001	0.616	< 0.001
Female	0.077	0.006	-	-	-	-
Age	-	-	0.113	0.002	-	-
More than five somatic diagnoses	-	-	−0.094	0.001	-	-
Number of mental disorders	-	-	-	-	−0.063	0.006
Not (only) German citizenship	−0.058	0.023	-	-	−0.087	0.001
Receiving a disability pension	−0.044	0.032	-	-	-	-
Standard rehabilitation in an expedited procedure	0.073	0.002	0.059	0.045	0.025	0.041
Adjusted R^2^	0.246		0.172		0.405	

Confounders to predict discharge scores of HOOS-PS, VR-12 PCS, and MCS with beta weights, coefficient p-values, and adjusted R^2^ for the respective model

“-“ = confounder not significant (*p* > 0.10) and not included in the final model for the respective outcome. Tested confounders not significant in any of the three models are omitted from the table

#### Objective (3)

Effect sizes of residuals (d_res_) derived from regression models showed for KOOS-PS range = 1.61 (− 0.52 to 1.09) and IQR = 0.33 (− 0.25 to 0.08); for VR-12 PCS range = 1.15 (− 0.51 to 0.64) and IQR = 0.41 (− 0.24 to 0.17); and for VR-12 MCS range = 0.96 (− 0.41 to 0.55) and IQR = 0.17 (− 0.10 to 0.07; Table 8). Figure 2 panels (d–f) present facility-level d_res_ with 95% CIs for facilities with *n* ≥ 15 (x-axis/zero line indicates no deviation from expected outcomes). Significant deviations from zero (per CIs) were evident for: KOOS-PS (V, D above; M, N below); VR-12 PCS (V, D above; K, A, N, M below); VR-12 MCS (V above; N below).

**Table 8:** Completion rates and effect sizes of residuals (d_res_) for KOOS-PS, VR-12 PCS, and MCS, and ProQI in TKA

	**All**	**KOOS-PS**			**VR-12 PCS**			**VR-12 MCS**			**TKA ProQI**		
**Facility**	**N**	**n**	**CR (%)**	**d**_**res**_	**n**	**CR (%)**	**d**_**res**_	**n**	**CR (%)**	**d**_**res**_	**n**	**CR (%)**	**ProQI**
A	49	46	94	− 0.29	44	90	− 0.36	47	96	0.01	43	88	83.2
B	59	34	58	0.18	37	63	0.20	36	61	− 0.08	34	58	92.1
C	30	6	20		8	27		8	27		6	20	
D	62	40	65	0.43	36	58	0.42	41	66	0.07	32	52	99.5
E	34	29	85	− 0.07	32	94	0.03	30	88	− 0.10	29	85	88.3
F	34	33	97	− 0.27	34	100	0.01	34	100	− 0.05	33	97	85.3
G	39	38	97	− 0.25	39	100	− 0.03	39	100	0.04	38	97	85.6
H	52	46	88	0.12	45	87	0.21	47	90	− 0.04	43	83	93.0
I	53	42	79	0.08	42	79	− 0.18	42	79	0.18	42	79	90.7
J	10	1	10		2	20		2	20		1	10	
K	38	33	87	− 0.34	33	87	− 0.35	33	87	− 0.16	33	87	82.3
L	45	30	67	− 0.06	19	42	0.24	31	69	0.13	19	42	91.1
M	69	28	41	− 0.39	28	41	− 0.51	28	41	0.15	27	39	82.4
N	40	36	90	− 0.52	38	95	− 0.37	38	95	− 0.41	36	90	76.9
O	43	40	93	0.04	40	93	0.07	41	95	− 0.06	39	91	90.5
P	35	34	97	− 0.02	35	100	− 0.12	35	100	0.13	34	97	90.1
Q	75	65	87	− 0.23	72	96	− 0.04	72	96	− 0.10	64	85	85.0
R	43	39	91	0.21	41	95	− 0.24	42	98	− 0.05	38	88	90.9
S	24	16	67	− 0.19	17	71	0.17	16	67	− 0.20	16	67	87.1
T	53	47	89	− 0.10	36	68	0.13	49	92	− 0.15	34	64	88.5
U	65	46	71	− 0.22	49	75	− 0.26	48	74	− 0.03	46	71	84.9
V	61	61	100	1.09	61	100	0.64	61	100	0.55	61	100	100.0
W	126	84	67	0.06	85	67	0.07	84	67	0.01	84	67	91.3
**Facility level**	**K = 23**	**k = 21**			**k = 21**			**k = 21**			**k = 21**		
Min	10	16	41	− 0.52	17	41	− 0.51	16	41	− 0.41	16	39	76.9
PCT 25	36.5	33	67	− 0.25	34	68	− 0.24	34	69	− 0.10	33	67	85.0
Median	45	39	87	− 0.07	38	87	0.01	41	90	− 0.04	36	85	88.5
PCT 75	60	46	93	0.08	44	95	0.17	47	96	0.07	43	90	91.1
Max	126	84	100	1.09	85	100	0.64	84	100	0.55	84	100	100.0
Range	116	68	59	1.61	68	59	1.15	68	59	0.96	68	61	23.1
IQR	23.5	13	26	0.33	10	27	0.41	13	27	0.17	10	23	6.1

Completion Rates: For the KOOS-PS, VR-12 PCS, and MCS, rates refer to cases with complete PROM scores and all required risk-adjustment variables (socio-demographic/medical) at both admission and discharge; the ProQI completion rate further requires full data availability across all three PROM scales simultaneously. d_res_ (Effect Size of Residuals): A risk-adjusted, scale-independent measure representing the difference between a patient’s observed discharge score and the value predicted by the regression model, expressed as a standardized effect size. ProQI (Patient-Reported Outcome Quality Index): A composite index (0–100) aggregating weighted, risk-adjusted residuals across disease-specific and generic health domains to benchmark facility performance. Upper section: Facility-specific results (n, completion rates (submitted data/sufficiently complete data), effect sizes of residuals (d_res_), composite Patient-reported Quality Index (ProQI)), including facilities with *n* < 15 cases. Lower section: Distributional summaries across facilities (k: number of facilities with *n* ≥ 15, min, max, median, quartiles, range, IQR), excluding facilities with *n* < 15 per prespecified criteria

#### Objective (4)

ProQI composite for TKA had a range = 23.1 (76.9 to 100) and IQR = 6.1 (85.0 to 91.1; Table 8; Supplementary Fig. S2), corresponding to Cohen’s d of 0.77 (range) and 0.20 (IQR) on the ProQI scale (M = 90, SD = 30). Due to insufficiently complete data, no ProQI could be calculated for facilities C and J.

#### Data completeness

Post-regression completeness rates were 41–100% for KOOS-PS (median = 87%; IQR: 67–93%; k = 21), 41–100% for VR-12 PCS (median = 87%; IQR: 68–95%; k = 21), and 41–100% for VR-12 MCS (median = 90%; IQR: 69–96%; k = 21; Table 8). Across all facilities, *n* = 825 cases yielded complete ProQI data, corresponding to an overall completion rate of 72% (median = 85%; range: 39–100%, IQR: 67–90%).

Subsequent non-completer analyses indicated that non-completers (cases for which no patient-level ProQI could be computed) differed slightly from completers in several baseline characteristics. They showed higher baseline PCS values (+ 2.24 points; *t* = − 3.666; df = 1067; *p* < 0.01; Supplementary Table S5), were on average 1.4 years older (*t* = − 1.972; df = 1063; *p* = 0.049; Supplementary Table S6), and less often diagnosed with mental disorders (− 3.1%; χ^2^ = 3.928; df = 1; *p* = 0.047). In addition, non-completers were less frequently employed at least part-time (− 11.8%; χ^2^ = 45.059; df = 11; *p* < 0.01) and reported work incapacity before rehabilitation less often (− 11.9%; χ^2^ = 11.624; df = 1; *p* < 0.01). Conversely, they more often participated in day-care rehabilitation (+ 2.4%; χ^2^ = 6.056; df = 1; *p* = 0.014; Supplementary Table S7). No significant differences in ProQI were found between patients from facilities with high and low completion rates (mean difference = 0.80; *t* = 0.487; df = 823; *p* = 0.627; Supplementary Table S8).

## Discussion

This pilot study aimed to utilize PROMs to evaluate and compare short-term outcomes of orthopedic rehabilitation facilities for post-THA or TKA patients, assess their discriminative power between facilities through risk-adjusted benchmarking, identify patient-level factors associated with outcomes, and determine the framework’s suitability for large-scale public reporting.

Twenty-three facilities participated in the pilot study and submitted data on included patients. As an important first step in quality control, we created an automated data validation report for each facility. This report identified any missing or implausible values in the transmitted dataset for each patient. The report allowed facilities to identify and, when possible, add or correct these values. This procedure aimed to increase the completion rate and to improve the overall reliability of the results.

After data validation, complete pre-post PROM scores were available for 86% (*n* = 961) of submitted THA cases (*N* = 1,107) and 84% (*n* = 961) of TKA cases (*N* = 1,139). Two facilities were excluded from the benchmarking analyses of d_z_ pre-post effect sizes, overall pooled mean d_z_ effect sizes, effect sizes d_res_ derived from risk-adjusted residuals, and the composite Patient-Reported Quality Index (ProQI) in the THA/TKA samples, as they fail to meet the minimum case criterion of *n* ≥ 15, which is considered necessary for representativeness and reliability. One additional facility was excluded from HOOS-PS and ProQI benchmarking in the THA sample for the same reason. Multiple regression models for risk adjustment required complete data on selected socio-demographic and medical variables, while ProQI calculations used combined risk-adjusted PROM results, excluding further cases with missing data. ProQI calculations across all facilities yielded complete data for *n* = 760 THA cases (69%; median 72%, range 28–100%) and *n* = 825 TKA cases (72%; median 85%, range 39–100%), reflecting the combined completeness requirements of all risk-adjusted PROMs. These rates reflect substantial data loss for facility-level analyses of the ProQI (31% THA, 28% TKA) and marked between-facility heterogeneity in data completeness, particularly pronounced in THA facilities. Primary reasons for missing data included incomplete PROM administration at admission or discharge, item-level non-response, and organizational or technical challenges in implementing the study protocol in some facilities. Patients occasionally reported certain items as inapplicable in the acute post-surgical setting, i.e., specific movements discouraged early after THA/TKA (see also [20]). This was particularly evident for HOOS-PS in the THA sample, where completeness was substantially lower than for VR-12 PCS/MCS. Patients possibly omitted unfeasible items (e.g., “running”) rather than rating “extreme difficulty”, selectively excluding acute postoperative floor effects from analyses. This may compromise HOOS-PS content validity in early rehabilitation and bias observed pre-post effect sizes, as collected data potentially include more responses from relatively healthier patients. Given postoperative medical restrictions persisting throughout rehabilitation, consistent “extreme difficulty” ratings across time points or selective non-response are more likely to underestimate observed HOOS-PS pre-post effect sizes.

### Data completeness

Non-completer analyses indicated limited patient-level selection. In THA, ProQI non-completers had slightly higher baseline VR-12 PCS scores. In TKA, non-completers showed similarly higher PCS, were slightly older, less often mentally diagnosed, less frequently employed, reported less pre-rehabilitation work incapacity, and more often attended day-care rehabilitation. However, ProQI scores did not differ significantly between patients from high- vs. low-completion facilities, indicating no systematic facility-level selection bias in our benchmarking results.

In line with ICHOM’s recommendations regarding a standard set of outcome measures, we used the short versions of the HOOS and KOOS [9]. Unlike the long forms, these contain only five and seven items, respectively, and only measure physical function as an outcome domain. Therefore, it is recommended that a more comprehensive generic quality-of-life measure be added, such as the VR-12. Our correlation analyses confirmed substantial overlap between specific and generic physical outcomes, with coefficients falling within the a priori range of 0.30 < *r* < 0.70 (*r* = 0.48–0.56 across admission/discharge and HOOS-PS/KOOS-PS with PCS) and explaining moderate amounts of common variance. This suggests that using a combination of specific and generic measures is appropriate.

Despite being significantly shorter than the original versions, the short versions are sufficiently reliable, valid, and sensitive to change during their development [12, 14], which is why they have been recommended. The main advantage of short forms is that they are more easily accepted by patients and practitioners in clinical settings due to their economical application. This could result in higher participation rates and more reliable data.

### Objective (1)

The broad range of scores across facilities at admission, along with considerable within-facility standard deviations, indicates that patients undergoing THA or TKA were able to evaluate their functional limitations and quality of life using the selected PROMs with sufficient nuance and discrimination between facilities. The substantial variability in admission scores and within-facility standard deviations was expected for the disease-specific (HOOS-PS, KOOS-PS) and the generic physical (VR-12 PCS) domains. However, it was also notably observed for the VR-12 mental component (MCS) domain. These results confirm the complementary selection of PROMs to capture biopsychosocial heterogeneity in orthopedic rehabilitation populations, enabling meaningful between-facility benchmarking across all domains. This heterogeneity also underscores the need for risk adjustment to distinguish genuine performance differences from case-mix variation.

At admission to rehabilitation, VR-12 PCS scores were markedly below the normative population mean of 50 (THA: 27.82; TKA: 27.84), while MCS scores were near the midpoint (THA: 47.60; TKA: 46.49), both expressed as T-scores with population norms of M = 50 and SD = 10. These values align well with preoperative baseline scores reported in other THA/TKA studies for PCS (e.g., [21–23) and MCS (e.g., [24]), indicating the representativeness of our rehabilitation population prior to the intervention period.

Observed pre-post effect sizes confirmed PROM sensitivity and discrimination. Unadjusted facility-level d_z_ distributions exceeded a priori discrimination thresholds (range ≥ 0.50, IQR ≥ 0.20) across all PROMs. All pooled sample effect sizes met or exceeded the a priori sensitivity threshold of d_z_ ≥ 0.40 (THA/TKA: d_z_ = 0.76/0.83 HOOS-PS/KOOS-PS, 0.76/0.71 PCS, 0.45/0.39 MCS), with ≥ 75% of facilities exceeding d_z_ ≥ 0.40 in physical domains (HOOS-PS/KOOS-PS, VR-12 PCS) vs. ≥ 50% in MCS. A few facilities per PROM showed non-significant pre-post changes (THA: HOOS-PS: 2 facilities; MCS: 5 facilities; TKA: PCS 2 facilities; MCS: 4 facilities). Complex orthopedic rehabilitation addresses psychological quality of life alongside physical function; unexpectedly robust MCS effect sizes also confirm VR-12 MCS sensitivity and discrimination, enabling feasible complementary use in studies and benchmarking of short-term rehabilitation outcomes in this specific population. Overall, these results demonstrate that the selected PROMs can detect change and distinguish between facilities. Therefore, they fulfil the prerequisites for subsequent risk-adjusted performance comparisons.

Remarkably, disease-specific PROMs showed only marginally higher effect sizes than generic VR-12 PCS in physical domains. Despite comparable effect sizes, specific and generic measures shared only 25% common variance, confirming their complementary necessity as recommended (e.g., [9]). Notably, moderate MCS improvements occurred despite admission scores near population norms. This could reflect the biopsychosocial approach of comprehensive rehabilitation, where physical gains drive mental health improvements and vice versa [25]. Alternatively, given that MCS scores were close to the norm and had lower effect sizes than physical domains, this could indicate that there are ceiling effects that limit psychological gains, or it could underscore the primacy of physical recovery in THA/TKA rehabilitation.

To the best of our knowledge, no previous studies have used PROMs to compare outcomes of a three-week complex rehabilitation program carried out shortly after surgery. Most studies report effect sizes for PROM comparisons between preoperative and follow-up assessments, typically within three to twelve months. For instance, Davis, Perruccio [26] reported higher standardized response means (SRMs, equal to Cohen’s d_z_) than we found: 1.50 for HOOS-PS and 1.40 for KOOS-PS at the six-month follow-up. In a large German sample, results 12 months after surgery also showed higher values, with standardized differences of 1.45 for HOOS-PS and 1.08 for KOOS-PS [23]. However, without follow-up data in this pilot study, we cannot empirically confirm the persistence or further progression of recovery trajectories beyond the immediate post-treatment period.

To assign clinical meaning to observed changes in PROM scores on the patient level, several studies have derived a minimally important clinical difference (MCID), defined as the smallest change perceived as relevant by patients (see [27]). For example, in two larger studies that calculated distribution-based MCIDs for THA and TKA on changes between pre-surgery and 1 yr follow-up, an MCID of 9.47 (range of the normalized scale: 0–100) was reported for HOOS-PS [28], and an MCID of 8.00 for KOOS-PS [29]. For VR-12 PCS/MCS, anchor-based MCIDs of 6.2 and 6.1 points were calculated in elective orthopedic patients at 2-month follow-up [30]. Using our study’s admission/discharge standard deviations and pre-post correlations, these MCIDs translate to d_z_ equivalents of 0.45 (HOOS-PS/KOOS-PS), 0.60 (VR-12 PCS), and 0.50 (VR-12 MCS), representing average clinically relevant improvements at the facility-level. However, since thresholds of the MCID and related statistical metrics, such as the minimal detectable change and the patient-acceptable symptom state (PASS), can vary widely depending on factors like the type of intervention, the preoperative diagnosis, the estimation method, and the nature of the anchor questions in the case of anchor-based estimations [28, 31], study-specific MCID equivalents derived from our rehabilitation samples provide more appropriate clinical benchmarks. Following the common distribution-based approach of calculating MCIDs as half the standard deviation of pre-post differences [32], this corresponds to a d_z_ equivalent of 0.50 across all PROM scales at the facility-level, yielding MCIDs of 11.18 (HOOS-PS), 8.82 (KOOS-PS), 5.10/4.85 (VR-12 PCS THA/TKA), and 6.26/5.78 (VR-12 MCS THA/TKA). Using d_z_ equivalents of 0.50 as study-specific clinical significance thresholds, 75% of THA facilities exceeded HOOS-PS benchmarks, 100% of TKA facilities met KOOS-PS criteria, 90%/67% exceeded VR-12 PCS benchmarks (THA/TKA), and 43%/33% met VR-12 MCS thresholds. These results demonstrate robust average clinical improvements across most facilities, particularly in the physical domain, while highlighting procedure- and domain-specific variability.

### Objective (2)

Regarding risk-adjusted benchmarking, we refrained from including all confounders identified in prior literature within this pilot study, due to varying data completeness of risk adjustment variables across facilities. This approach was intended to ensure a reasonably high number of cases at each facility, a sufficient number of facilities for evaluation, and confidence intervals small enough to enable meaningful comparisons between facilities. However, this approach may not adequately explain a significant proportion of the variance in outcomes. This would limit the fairness of the intended comparison. Non-completer analyses suggested minimal patient-level differences between completers and non-completers, and no systematic facility-level discrepancies in risk-adjusted ProQI scores. Nonetheless, as measuring outcome quality in orthopedic rehabilitation becomes part of routine clinical practice in the future, it is recommended that methods be developed to reliably and completely collect the necessary risk adjustment data. This will maximize the quality of risk adjustment and enable fair, unbiased, and valid results of adjusted benchmarking.

Across THA/TKA models, admission PROM scores emerged as the dominant predictors exhibiting the highest beta weights for all PROMs and explaining the largest proportion of outcome variance. The final multivariable models retained these baseline scores as well as female sex, age, high school degree, work incapacity at admission, physical and mental comorbidity, citizenship, disability pension, and expedited standard rehabilitation. The predictor pools were identical across THA/TKA, though the significance was procedure- and outcome-specific. These results confirm that baseline impairment is the primary risk adjustment factor in early postoperative rehabilitation. This comprehensive risk-adjustment approach, therefore, explicitly accounts for the considerable heterogeneity in admission scores observed across facilities. By accounting for these baseline differences, the models disentangle genuine performance variation from case-mix effects and mitigate the influence of regression-to-the-mean phenomena. Consequently, benchmarking results more accurately reflect facility-level outcome quality rather than variability in patient starting points.

Our risk adjustment models demonstrated modest explanatory power typical for PROM-based arthroplasty benchmarking, where baseline functional status consistently emerges as the dominant predictor across THA and TKA cohorts. This aligns with established literature on THA/TKA outcome prediction, where admission PROMs alone often capture 15–30% of discharge variance, with clinical factors such as BMI or ASA grade contributing only small standardized beta coefficients (typically β < 0.10) beyond baseline adjustment [33–35]. The residual 59.5–85.6% unexplained variance in this pilot study is considered to be primarily driven by between-facility-level variance rather than unmeasured patient factors and precisely defines the performance signal essential for meaningful benchmarking. Conceptually, high-R^2^ models would paradoxically suggest outcomes are largely predetermined by case mix, leaving minimal room to detect genuine quality differences between facilities. While omitted variables such as surgeon experience, implant method, and type may incrementally refine predictions, their marginal beta contributions in PROM-adjusted models are unlikely to substantially alter facility rankings, thereby preserving fairness in relative comparisons. However, there may be confounders not included that potentially contribute to a higher amount of explained variance, like early mobilization after surgery [33]. Furthermore, as information on the extent of patient assistance during PROM completion was not available, differences in data collection procedures across facilities—including varying modes of administration and patient support—may have introduced measurement bias, which may have contributed to residual variance beyond true performance differences [36]. Although systematic measurement biases across facilities remain a concern, their dilution within large pooled samples supports robust estimation of expected outcome values for benchmarking purposes. This interpretation is supported by the systematic and consistent estimation procedures applied across all facilities, making it more plausible that the remaining unexplained variance reflects genuine institutional differences in rehabilitation processes rather than random measurement error or omitted individual-level factors.

### Objective (3)

Risk-adjusted residual effect sizes (d_res_) demonstrated robust facility discrimination. Across all PROMs and facilities, d_res_ ranges, and IQRs met or substantially exceeded a priori discrimination thresholds (range ≥ 0.50, IQR ≥ 0.20), confirming sufficient statistical power to differentiate facility performance beyond case-mix variation. Visual inspection of the forest plots identified multiple significant facilities above and below the expected performance range (95% CIs excluding the zero-line indicating expected case-mix-adjusted performance: THA 2–4 facilities above, 0–3 below; TKA 1–2 above, 1–4 below), with two facilities consistently exceeding expected outcomes and variable facilities falling short across domains. Notably, moderate IQRs confirm that most facilities show case-mix-adjusted outcomes close to expectations, indicating broadly comparable rehabilitation quality, with outliers driving range variability. These distributions validate d_res_ as a reliable metric for case-mix-adjusted benchmarking, enabling identification of genuine risk-adjusted performance differences while facility-specific confidence intervals appropriately reflect sampling uncertainty.

Although single-level regression models are widely applied in benchmarking contexts [37], Hierarchical Linear Models (HLM) are theoretically preferred for clustered data as they correct standard errors for intra-facility correlation, whereas single-level OLS may underestimate standard errors and produce artificially narrow confidence intervals. However, for the effect sizes of residuals (d_res_), the forest plots revealed extensive 95% CI overlap among most facilities (only 10–33% of facilities per PROM had CIs excluding zero), yielding conservative inference patterns unlikely inflated by underestimated standard errors. Critically, in the THA sample, facilities with case volumes *n* < 30 maintained more extreme rankings from unadjusted (d_z_) to risk-adjusted (d_res_) forest plots, with modest rank changes. This rank stability across adjustment levels indicates genuine facility performance differences rather than sampling artifacts, validating the OLS residuals for benchmarking despite theoretical clustering concerns. Moreover, visual comparison of unadjusted pre–post effect sizes (d_z_) and risk-adjusted residual effect sizes (d_res_) across facilities revealed highly consistent patterns. Facilities that appeared as high performers in the unadjusted plots tended to occupy the upper ranks in the risk-adjusted plots, whereas low-performing facilities remained at the lower end of both distributions. This left-to-right gradient in point estimates, observable across all PROMs, supports the interpretation that our residual-based benchmarks capture genuine performance differences rather than model-induced artifacts, even in the presence of heterogeneous case volumes.

### Objective (4)

The Patient-Reported Outcome Quality Index (ProQI) combines complex risk-adjusted PROM results into a single, interpretable metric for public benchmarking. Constructed through expert consensus with German Pension Insurance representatives, clinicians, and researchers, ProQI weights disease-specific PROMs at 50% and VR-12 PCS/MCS each at 25% to reflect rehabilitation’s biopsychosocial model while balancing specific and generic health domains. This normative scheme prioritizes conceptual coherence over statistical optimization. However, future studies could explore weightings emphasizing physical domain dominance, disease-specific priority, or PROM reliability.

The 50/25/25 weighting scheme aligns with multimodal rehabilitation’s theoretical framework. Equal PCS/MCS weighting (25% each) acknowledges mental health as integral despite smaller MCS effect sizes, while the normative M = 90, SD = 30 scale incentivizes performance through its high reference point and enables facility differentiation via a 30-point spread. Upper capping at 100 stabilizes interpretability, given empirical distributions nearly all falling below this threshold. The choice of a normative mean of 90 and a standard deviation of 30 was a deliberate design decision to ensure the practical acceptance of the ProQI in a public reporting context. By setting a high reference point, the index avoids the negative connotations of low 'raw’ scores, focusing instead on positive differentiation. Furthermore, the SD of 30 ensures that even modest risk-adjusted differences (d_res_) still translate into meaningful point spreads, which is essential for stakeholders to distinguish between facilities in this reporting framework.

ProQI explicitly measures relative facility performance, not absolute clinical improvement. It quantifies deviations from case-mix-adjusted expectations rather than patient-level gains or MCID achievement, enhancing transparency when paired with PROM-specific analyses. MCS inclusion may modestly reduce physical-domain sensitivity given its relatively lower discriminative power, yet this normative decision comprehensively reflects rehabilitation objectives beyond isolated physical function while maintaining index stability.

ProQI distributions demonstrated robust discrimination capacity. THA results showed range = 24.6 (75.4–100), IQR = 8.0 (84.7–92.7); TKA range = 23.1 (76.9–100), IQR = 6.1 (85.0–91.1), corresponding to THA/TKA between-facility effect sizes of d = 0.77/0.82 (range spanning minimum–maximum facilities) and d = 0.20/0.27 (IQR spanning central 50% of facilities) relative to ProQI’s normative SD = 30. Moderate IQRs confirm most facilities cluster near expected risk-adjusted performance with clearly distinguishable facilities at distribution extremes, fulfilling benchmarking objectives.

ProQI advances practical risk-adjusted rehabilitation benchmarking. Its intuitive 0–100 scale facilitates stakeholder communication and public reporting while preserving methodological rigor, enabling identification of genuine performance variation beyond case-mix differences. When implemented, ProQI should be complemented with unadjusted effect sizes (d_z_) and risk-adjusted residual effect sizes (d_res_) to provide stakeholders with a comprehensive view encompassing both raw treatment changes and case-mix-adjusted facility performance. Additionally, longitudinal evaluation across multiple annual cohorts should empirically validate ProQI scaling parameters (M = 90, SD = 30) to ensure long-term appropriateness as benchmarking data accumulate. ProQI crucially depends on complete PROM and socio-demographic/medical covariate data; our study experienced ~ 30% data loss in the pooled samples and up to 70% in specific facilities, despite non-completer analyses suggesting negligible impact on ProQI. Complete data remain essential to prevent potential bias, underscoring the need for future implementations to minimize patient and provider non-response.

Facility-level visual inference revealed power limitations from insufficient case numbers. With too few patients per facility, 95% confidence intervals were often too wide to differentiate unadjusted effect sizes (d_z_) from significant deviations from the pooled average effect sizes, or risk-adjusted residuals (d_res_) from deviations from the expected case-mix outcomes. This limits the reliability of their benchmarking results when higher effect sizes cannot be confirmed as statistically significant above average or expectation [38]. A priori power analyses indicated *n* ≥ 50 complete cases per facility suffice for 80% power (α = 0.05) to detect d_z_ ≥ 0.40 across PROMs and confounders. If completeness rates observed in this study cannot be improved beyond current levels and given inter-facility completeness variability, facilities should target consecutive inclusion of ≥ 140 cases to ensure 80% of participating facilities achieve *n* = 50 complete cases, potentially requiring extended recruitment periods for robust, representative benchmarking.

### Strengths and limitations

This study presents a comprehensive framework for measuring and benchmarking patient-reported outcomes (PROMs) in orthopedic rehabilitation throughout Germany. Integrating PROMs into quality assurance processes reflects the growing emphasis on patient-centered evaluations in rehabilitation and broader health services research [1, 36]. While the approach demonstrated several methodological and practical strengths, it also revealed important limitations that must be considered when interpreting the findings.

A key strength lies in the selection and implementation of internationally recommended standardized PROMs. These instruments were chosen in collaboration with participating facilities and are based on established standards, including those of the International Consortium for Health Outcomes Measurement [9, 39]. This approach ensures both clinical relevance and international comparability. Using both generic and condition-specific PROMs enables a more detailed evaluation of patient outcomes by capturing changes in functional status and quality of life with high sensitivity [40]. The observed effect sizes, which, on average, particularly in the physical domains, reflect clinically meaningful improvement at the group level in the pooled samples and in most facilities, coupled with the ability to distinguish institutional performance through residual-based effect sizes and the ProQI, underscore the utility of PROMs for rehabilitation benchmarking and, in the long term, improving treatment quality [41].

Importantly, the study benefited from the participation of a large and heterogeneous group of facilities, which enhanced the representativeness and generalizability of the findings at the national level. The broad implementation across different institutional settings reflects the feasibility of incorporating PROMs into routine practice, which is consistent with the results of other initiatives [42].

The flexible, multimodal approach to data collection, which accommodated digital tools, paper-based surveys, and integration with hospital information systems, allowed institutions to adapt the methodology to their local contexts. While this likely contributed to acceptable data completeness and patient engagement, variations in implementation and associated patient support may have introduced measurement inconsistencies and could therefore have influenced the results to a limited extent [43].

Rigorous data quality assurance procedures, including institution-specific validation reports and iterative completeness checks, enhanced dataset credibility and reliability. However, as a pilot study embedded in routine clinical workflows, we could not systematically verify patient inclusion or calculate response rates, nor capture information on distributed questionnaires. On-site audits, while informative, were infeasible given available resources. Furthermore, the institutions stated that a key requirement for their participation was that the additional effort required for the study within their clinical routine had to be kept to a minimum.

Applying a comprehensive risk adjustment model that incorporated a broad set of confounders routinely utilized by the German Pension Insurance (DRV) provided a robust methodological foundation for fair institutional comparisons [44, 45]. Using effect sizes to describe observed change, combined with risk-adjusted comparisons, aligns with best practices in outcomes research and allows for the identification of meaningful over- or under-performance [46]. The development of the ProQI index, a simplified composite measure for public reporting, improves the accessibility of complex data, facilitating its broader use in quality management and user transparency initiatives, ideally without reducing too much of the underlying power of the information [23, 47, 48].

Furthermore, the benchmarking framework promotes institutional transparency and data verification. In accordance with quality management principles, all participating facilities received detailed individual reports and were granted access to verify their facility-specific data upon request. This feedback loop ensures that institutions can validate their performance metrics against the collective sample, establishing a necessary foundation for future quality development.

However, there are several limitations that must be discussed. First, although the selected PROMs are validated and accepted internationally, they may not capture all aspects of individual rehabilitation goals or the full scope of everyday clinical practice. Additionally, as some patients in this pilot study have pointed out, some items refer to everyday situations or assume that the patient has not recently undergone surgery. Consequently, some items may seem inappropriate in a hospital setting, despite scoring well within the overall scale. These issues raise concerns about construct and content validity in specific cases and leave room for further adjustment of the measures [49].

Second, as this study evaluates outcomes solely at discharge (approximately five to six weeks post-surgery) without long-term follow-up data, the findings should be interpreted as benchmarks for the immediate, short-term rehabilitation response [50]. While assessing performance at discharge is a critical metric for monitoring the intensive early recovery phase, future research incorporating follow-up data at six or twelve months is valuable. Such longitudinal data are required to examine whether these initial institutional differences remain stable or diminish over time and to determine the sustainability of the observed gains beyond the immediate rehabilitation setting.

Third, although the risk adjustment process accounted for a wide range of variables, residual confounding remains a possibility. Not all relevant factors may have been measured or coded consistently across institutions, particularly with regard to ICD diagnoses, which may affect the comparability of adjusted outcomes. Other relevant variables not included may consist of patient characteristics such as previous hip or knee operations, ASA score, body mass index, smoking and alcohol use, as well as a range of surgical factors such as surgical approach, type of prosthesis, and surgeon experience [33, 51, 52]. Results of these predictors are not always congruent, e.g., the plane of the surgical approach did not predict an increase in PROMs at the 6-month follow-up after THA in a large multicenter trial with 26 hospitals in North America [53].

Fourth, there are several limitations of the single-level modeling approach. OLS assumes observation independence, likely underestimating standard errors due to intra-facility correlation, particularly in smaller facilities (*n* = 16–30 comprising > 25% of THA and < 20% in TKA centers). This may narrow confidence intervals, though the observed pattern of extensive overlap among mid-range facilities produces conservative inference that minimizes Type I error inflation. Rank consistency between unadjusted and adjusted extremes further supports result robustness against pure sampling variability. With 21 facilities, HLM random effect variance estimates would have limited precision for facility discrimination [54], though cluster-robust standard errors represent a practical alternative for future analyses. Facility case volumes ≥ 50 would enhance ranking stability across modeling approaches. While several facilities reached statistical significance with 95% confidence intervals just excluding zero, these effects should be interpreted cautiously, given the combination of clustered data and variable case numbers. However, the consistent alignment of unadjusted and adjusted rankings and the apparent gradient of point estimates across facilities argue against these findings being solely driven by chance or model misspecification.

Fifth, we calculated Cohen’s d_z_, i.e., we divided the average pre-post difference by the standard deviation of the individual differences (SD_diff_) per facility. When calculating SD_diff_, the variability of the difference scores depends on SD_pre_ and SD_post_, as well as the correlation between the pre- and post-measures. Firstly, large values of SD_pre_ and SD_post_ increase SD_diff_, which can decrease Cohen’s d_z_ for a given mean difference, conversely less variability tends to increase Cohen’s d_z_. Secondly, higher correlations typically result in smaller SD_diff_ values, leading to larger d_z_ values for the same mean difference. This means that the level of the effect size for each facility is influenced by these three parameters, which may differ considerably between facilities. In line with others, we opted for this widely recommended approach because we consider the uniformity or consistency of change per facility (improvement or deterioration) as measured by correlation to be an important indicator of outcome quality [55, 56].

Sixth, as a relative performance metric, the ProQI quantifies how a facility performs compared to its case-mix-adjusted expectation, rather than measuring whether patients reached a specific clinical threshold (e.g., MCID). While this limits its utility for assessing absolute clinical improvement in e.g., decision-making context [57], it is a strengths-based approach for benchmarking, as it separates facility-level performance differences from patient-level case-mix differences. To provide a holistic view, we recommend that future public reports present the ProQI alongside unadjusted pre-post effect sizes to maintain the link between relative performance and absolute clinical gains. While our framework identifies significant performance differences between facilities, it does not currently capture data on structural or process quality. Consequently, the specific drivers behind a facility’s over- or under-performance—such as staffing levels, clinical pathways, or specific therapeutic interventions—remain unknown. While the ProQI provides a robust signal for identifying quality variations, it cannot yet explain the underlying causes, highlighting the need for future research to link these outcome-based benchmarks with detailed process and structural indicators.

Seventh, we encountered significant data incompleteness, with an overall attrition rate of approximately 30% in the pooled samples, reaching up to 70% in individual facilities. Additionally, the lack of access to consecutive patient logs precluded a formal non-responder analysis at recruitment. While these factors underscore the inherent challenges of implementing PROMs in routine clinical practice, our non-completer analyses demonstrated that the risk of systematic bias remains low. ProQI scores did not significantly differ between facilities with high (≥ 75%) and low (< 75%) completion rates, suggesting that the index remains a robust metric even under real-world conditions with suboptimal data completeness—provided the underlying risk-adjustment model is comprehensively specified. Nevertheless, we cannot entirely rule out a selection bias, and future large-scale implementations should prioritize standardized, consecutive enrollment monitoring to ensure full representativeness and minimize potential bias.

Eighth, this evaluation was observational, without a randomized control group. Consequently, the observed PROM changes cannot be interpreted as evidence of causal effectiveness [58].

## Conclusions

The pilot study shows that PROMs can be effectively implemented on a large scale in complex orthopedic rehabilitation programs. When combined with comprehensive risk adjustment and robust quality control measures, PROMs can be used to compare institutional performance. The developed framework enables meaningful benchmarking and lays the groundwork for transparent, outcome-oriented quality management. By integrating both generic and disease-specific dimensions, our framework captures the biopsychosocial complexity of multimodal rehabilitation, moving beyond purely functional assessments. Although limitations regarding data comparability and causal inference exist, this approach is a significant step toward patient-centered, evidence-based evaluation in rehabilitation care. Thus, this pilot study paves the way for the large-scale use of PROMs as outcome-quality benchmarks from the patient’s perspective in orthopedic rehabilitation.

Integrating PROMs into routine rehabilitation care offers tangible benefits for healthcare providers, patients, and policymakers. Providers can use PROM-based benchmarking to initiate processes that identify their institutions’ strengths and areas for improvement. Public reporting through simplified indices like ProQI fosters accountability and may incentivize quality improvement. For patients, PROMs highlight their perspective, aligning care more closely with their priorities and perceived progress. Furthermore, policymakers and insurers can use the data to make evidence-based decisions about resource allocation and system performance.

However, maximizing these benefits requires greater efforts to standardize PROM implementation procedures across institutions, ensure comprehensive confounder measurement, and support the clinical interpretation of benchmarking outcomes.

Further research should explore several critical areas. First, including additional outcome domains, such as patient experience, return to work, and long-term follow-up, could provide a more comprehensive view of rehabilitation success. Second, studies should investigate the relationship between structural and process variables (e.g., staffing, therapy intensity, and multidisciplinary coordination) and outcome variability. Linking outcome data with contextual information could help explain observed differences and inform targeted quality improvements.

Additionally, future efforts should seek to refine the ProQI composite measure to reflect clinically meaningful thresholds, improve interpretability, and potentially integrate machine learning models for predictive analytics. Longitudinal studies and randomized controlled trials that incorporate PROMs as primary endpoints would also help establish causal relationships between interventions and patient-perceived benefits in rehabilitation.

## Supplementary Information

Supplementary Material 1: Fig. S1. Results of the Patient-reported Outcome Quality Index across 21 rehabilitation facilities treating THA patients. Fig. S2. Results of the Patient-reported Outcome Quality Index across 21 rehabilitation facilities treating TKA patients. Tables S1-S4: Detailed results of the non-completer analysis for total hip arthroplasty (THA). Tables S5-S8: Detailed results of the non-completer analysis for total knee arthroplasty (TKA).

## Data Availability

The datasets used and/or analyzed during the current study are available from the corresponding author on reasonable request.
