# Supplementary material for: Differences in health outcomes of orthopedic rehabilitation after hip or knee replacement: a prospective pilot study benchmarking 23 rehabilitation facilities using Patient-Reported Outcome Measures (PROMs)
Source: Arthroplasty. 2026 Aug 3;8:55. doi: 10.1186/s42836-026-00401-x (PMC13430887; doi:10.1186/s42836-026-00401-x)
Supplement: Supplementary file 1 — Supplementary Material 1: Fig. S1. Results of the Patient-reported Outcome Quality Index across 21 rehabilitation facilities treating THA patients. Fig. S2. Results of the Patient-reported Outcome Quality Index across 21 rehabilitation facilities treating TKA patients. Tables S1-S4: Detailed results of the non-completer analysis for total hip arthroplasty (THA). Tables S5-S8: Detailed results of the non-completer analysis for total knee arthroplasty (TKA). [file 42836_2026_401_MOESM1_ESM.docx]

**Supplementary Figures**


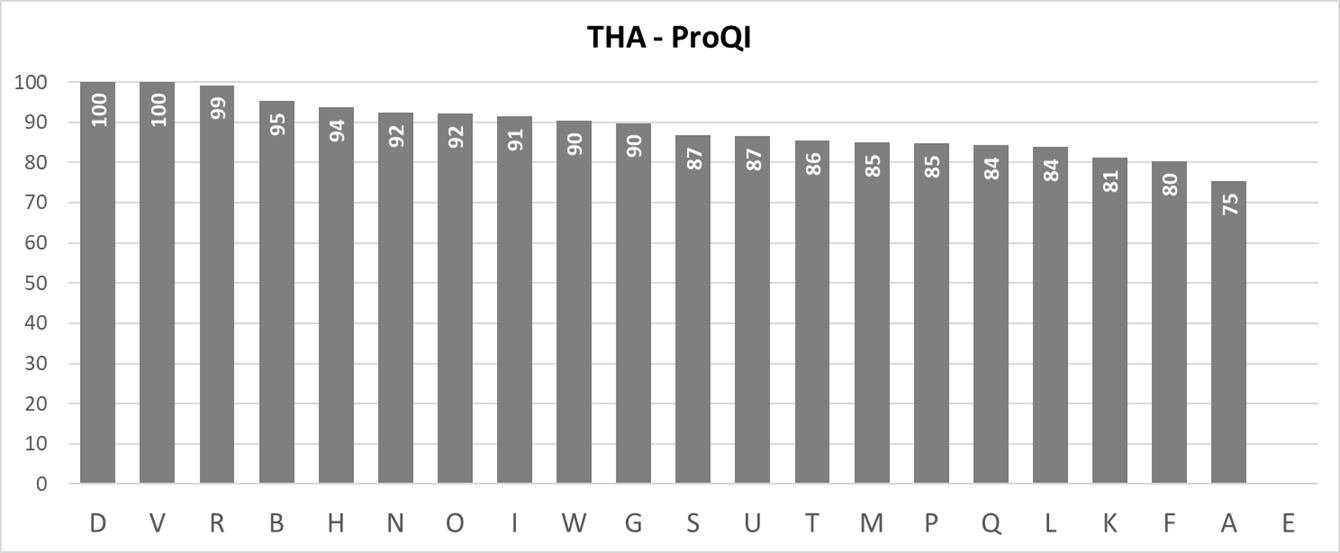


**Fig. S1**. Results of the Patient-reported Outcome Quality Index (ProQI: composite index visualizing risk-adjusted performance differences between facilities, based on weighted standardized residuals of expected vs. observed PROM outcomes for HOOS-PS and VR-12 PCS/MCS) across 21 rehabilitation facilities treating THA patients. Due to insufficient data completeness, no ProQI could be calculated for facility E; an empty data point was retained for consistency with other manuscript figures.


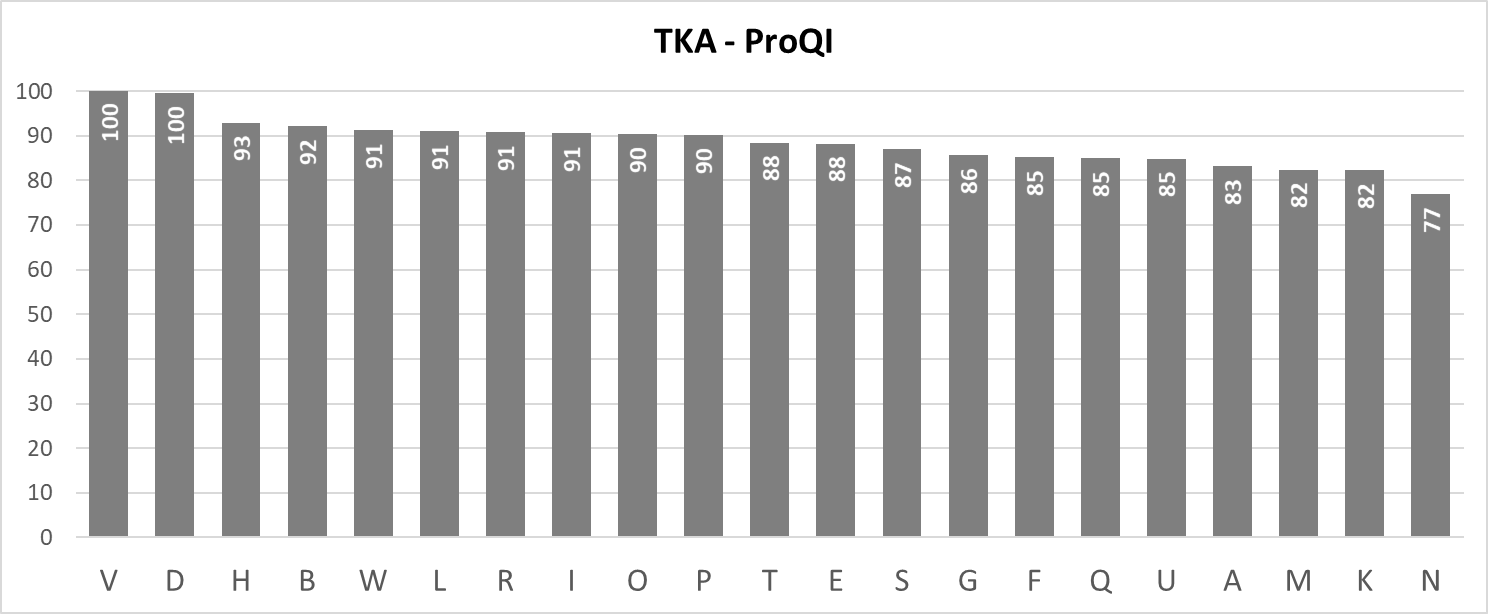


**Fig. S2**. Results of the Patient-reported Outcome Quality Index (ProQI: composite index visualizing risk-adjusted performance differences between facilities, based on weighted standardized residuals of expected vs. observed PROM outcomes for KOOS-PS and VR-12 PCS/MCS) across 21 rehabilitation facilities treating TKA patients.

**Detailed results of the non-completer analysis and ProQI score differences between high and low data completeness facilities**

To evaluate potential selection bias and the robustness of the facility-level benchmarking, a non-completer analysis was performed separately on the pooled samples of the Total Hip Arthroplasty (THA) and Total Knee Arthroplasty (TKA) sub-groups. Patients for whom a ProQI could be calculated (“completers”)—requiring full data availability across all three PROM scales at admission and discharge, and all socio-demographic and medical risk-adjustment variables—were compared against those with missing data ("non-completers").

Statistical comparisons were conducted using independent-samples t-tests for metric variables, such as PROM scores and age at admission (T0) and at discharge (T1). Chi-square (Χ²) tests were utilized for categorical variables, including sex, marital status, employment status, and clinical characteristics. Finally, to assess whether data quality influenced performance outcomes, the ProQI scores of patients in facilities with high data completeness (≥75%) were compared with those in facilities with lower completeness (<75%) using independent-samples t-tests. For all analyses, differences between groups were considered statistically significant when the null hypothesis could be rejected at a significance level of *p* < 0.05.

**Results of the non-completer analysis for total hip arthroplasty (THA)**

**Table S1.** Comparison of admission and discharge PROM scores between completers and non-completers (THA).

| **Scale** | **Group** | ***n*** | **M (SD)** | **M_diff_** | ***t*** | **df** | ***p*** |
| --- | --- | --- | --- | --- | --- | --- | --- |
| HOOS-PS t0 | Completers | 779 | 45.17 (21.07) | −1.35 | −0.832 | 990 | 0.406 |
|  | Non-Completers | 213 | 46.52 (20.37) |  |  |  |  |
| HOOS-PS t1 | Completers | 779 | 62.56 (17.70) | 2.29 | 1.356 | 911 | 0.175 |
|  | Non-Completers | 134 | 60.27 (20.13) |  |  |  |  |
| **VR-12 PCS t0** | **Completers** | **779** | **27.44 (8.66)** | **−2.04** | **−3.305** | **1048** | **<0.001** |
|  | **Non-Completers** | **271** | **29.48 (9.03)** |  |  |  |  |
| VR-12 PCS t1 | Completers | 779 | 35.47 (8.40) | −0.64 | −1.001 | 1003 | 0.317 |
|  | Non-Completers | 226 | 36.10 (8.42) |  |  |  |  |
| VR-12 MCS t0 | Completers | 779 | 47.63 (13.73) | 1.78 | 1.807 | 1032 | 0.071 |
|  | Non-Completers | 271 | 45.85 (13.38) |  |  |  |  |
| VR-12 MCS t1 | Completers | 779 | 53.20 (12.05) | 1.16 | 1.237 | 996 | 0.216 |
|  | Non-Completers | 219 | 52.04 (12.99) |  |  |  |  |

Independent t-test results comparing HOOS-PS, VR-12 PCS, and VR-12 MCS scores at admission (T0) and discharge (T1) for the THA sub-sample.

**Table S2.** Comparison of mean age between completers and non-completers (THA).

| **Characteristic** | **Group** | ***n*** | **M (SD)** | **M_diff_** | ***t*** | **df** | ***p*** |
| --- | --- | --- | --- | --- | --- | --- | --- |
| Age | Completers | 777 | 67.35 (11.01) | −1.03 | −1.337 | 1048 | 0.182 |
|  | Non-Completers | 273 | 68.38 (10.80) |  |  |  |  |

Independent t-test results for age (years) between the completer and non-completer groups in the THA sub-sample.

**Table S3.** Comparison of categorical socio-demographic, clinical, and rehabilitation characteristics between completers and non-completers (THA).

| **Characteristic** | **Category** | **Completers** | | **Non-Completers** | | **Χ²** | **df** | ***p*** |
| --- | --- | --- | --- | --- | --- | --- | --- | --- |
|  |  | ***n*** | **%** | ***n*** | **%** |  |  |  |
| Sex | Female | 431 | 55.3 | 161 | 59.2 | 1.224 | 1 | 0.269 |
|  | Male | 348 | 44.7 | 111 | 40.8 |  |  |  |
| Only German nationality | No | 9 | 1.2 | 6 | 2.2 | 1.471 | 1 | 0.225 |
|  | Yes | 764 | 98.8 | 270 | 97.8 |  |  |  |
| Marital status | Single | 59 | 7.6 | 17 | 6.3 | 7.536 | 5 | 0.184 |
|  | Married | 485 | 62.3 | 167 | 61.9 |  |  |  |
|  | Seperated | 11 | 1.4 | 2 | 0.7 |  |  |  |
|  | Divorced | 76 | 9.8 | 17 | 6.3 |  |  |  |
|  | Widowed | 144 | 18.5 | 66 | 24.4 |  |  |  |
|  | Remarried | 4 | 0.5 | 1 | 0.4 |  |  |  |
| Current committed relationship | No | 198 | 31.6 | 55 | 29.9 | 0.189 | 1 | 0.664 |
|  | Yes | 429 | 68.4 | 129 | 70.1 |  |  |  |
| Highest school degree | In school (enrolled) | 1 | 0.1 | 1 | 0.4 | 5.019 | 6 | 0.541 |
|  | No degree | 8 | 1.0 | 5 | 2.0 |  |  |  |
|  | Special ed. degree | 1 | 0.1 | 1 | 0.4 |  |  |  |
|  | Basic secondary | 329 | 42.2 | 104 | 42.4 |  |  |  |
|  | Intermediate secondary | 249 | 32.0 | 84 | 34.3 |  |  |  |
|  | High school/ Academic secondary | 157 | 20.2 | 43 | 17.6 |  |  |  |
|  | Other | 34 | 4.4 | 7 | 2.9 |  |  |  |
| Current employment/ income status | Employed, full-time | 158 | 20.5 | 47 | 18.5 | 14.155 | 11 | 0.225 |
|  | Employed, part-time | 49 | 6.4 | 16 | 6.3 |  |  |  |
|  | Marginally employed | 5 | 0.7 | 4 | 1.6 |  |  |  |
|  | Unpaid family worker | 4 | 0.5 | 4 | 1.6 |  |  |  |
|  | Homemaker | 32 | 4.2 | 15 | 5.9 |  |  |  |
|  | Unemployed (registered) | 24 | 3.1 | 6 | 2.4 |  |  |  |
|  | Permanent disability | 13 | 1.7 | 8 | 3.1 |  |  |  |
|  | Temporary disability | 6 | 0.8 | 4 | 1.6 |  |  |  |
|  | Retired | 408 | 53.1 | 120 | 47.2 |  |  |  |
|  | Survivor's benefits | 63 | 8.2 | 29 | 11.4 |  |  |  |
|  | Other not in labor force | 5 | 0.7 | 1 | 0.4 |  |  |  |
|  | Unknown/not specified | 2 | 0.3 | 0 | 0.0 |  |  |  |
| Work incapacity at admission | No | 585 | 77.7 | 158 | 74.9 | 0.735 | 1 | 0.391 |
|  | Yes | 168 | 22.3 | 53 | 25.1 |  |  |  |
| Pending disability claim | No | 615 | 95.9 | 147 | 95.5 | 0.075 | 1 | 0.785 |
|  | Yes | 26 | 4.1 | 7 | 4.5 |  |  |  |
| Setting of rehabilitation | Inpatient | 758 | 98.6 | 309 | 99.0 | 0.382 | 1 | 0.537 |
|  | Day-care | 11 | 1.4 | 3 | 1.0 |  |  |  |
| Standard rehabilitation in an expedited procedure | No | 673 | 93.7 | 264 | 94.3 | 0.107 | 1 | 0.743 |
|  | Yes | 45 | 6.3 | 16 | 5.7 |  |  |  |
| Side of surgery | Left | 332 | 43.3 | 144 | 46.2 | 0.868 | 2 | 0.648 |
|  | Right | 407 | 53.1 | 159 | 51.0 |  |  |  |
|  | Both sides | 27 | 3.5 | 9 | 2.9 |  |  |  |
| More than five somatic diagnoses | No | 638 | 81.9 | 210 | 79.2 | 0.914 | 1 | 0.339 |
|  | Yes | 141 | 18.1 | 55 | 20.8 |  |  |  |
| One or more mental disorders | No | 730 | 93.7 | 304 | 93.0 | 0.209 | 1 | 0.647 |
|  | Yes | 72 | 6.5 | 23 | 7.0 |  |  |  |

Chi-square test results for sex, citizenship, marital status, education level, employment status, work incapacity, disability claims, rehabilitation setting, surgery side, and comorbidity for the THA sub-sample.

**Table S4.** Comparison of ProQI scores between high and low data completeness facilities (THA).

| **Outcome** | **Patients in facilities with Completion rate (CR ≥ 75% vs. CR < 75%)** | ***n*** | **M (SD)** | **M_diff_** | ***t*** | **df** | ***p*** |
| --- | --- | --- | --- | --- | --- | --- | --- |
| ProQI THA ^c^ | CR ≥ 75% ^a^ | 395 | 89.10 (22.96) | −1.73 | −1.050 | 758 | .294 |
|  | CR < 75% ^b^ | 365 | 90.82 (22.32) |  |  |  |  |

Independent t-test comparing the Patient-Reported Outcome Quality Index (ProQI) between facilities with completion rates ≥ 75% and those with < 75% for THA.

a: Facilities A, F, G, H, I, K, P, Q, S, V.

b: Facilities B, D, L, M, N, O, R, T, U, W.

c: Due to insufficient case numbers (*n* < 15) for facilities C, E & J, no ProQI could be calculated.

**Results of the non-completer analysis for total knee arthroplasty (TKA)**

**Table S5.** Comparison of admission and discharge PROM scores between completers and non-completers (TKA).

| **Scale** | **Group** | ***n*** | **M (SD)** | **M_diff_** | ***t*** | **df** | ***p*** |
| --- | --- | --- | --- | --- | --- | --- | --- |
| KOOS-PS t0 | Completers | 832 | 47.44 (17.82) | −2.40 | −1.772 | 1045 | 0.077 |
|  | Non-Completers | 215 | 49.84 (17.31) |  |  |  |  |
| KOOS-PS t1 | Completers | 832 | 62.38 (13.13) | −1.27 | −1.118 | 999 | 0.264 |
|  | Non-Completers | 169 | 63.64 (14.84) |  |  |  |  |
| **VR-12 PCS t0** | **Completers** | **832** | **27.55 (8.21)** | **−2.24** | **−3.666** | **1067** | **<0.001** |
|  | **Non-Completers** | **237** | **29.79 (8.56)** |  |  |  |  |
| VR-12 PCS t1 | Completers | 832 | 34.68 (8.07) | −0.61 | −0.912 | 1007 | 0.362 |
|  | Non-Completers | 177 | 35.29 (8.06) |  |  |  |  |
| VR-12 MCS t0 | Completers | 832 | 46.44 (14.21) | −0.33 | 0.313 | 1063 | 0.754 |
|  | Non-Completers | 233 | 46.12 (13.72) |  |  |  |  |
| VR-12 MCS t1 | Completers | 832 | 50.78 (12.52) | −1.14 | −1.093 | 1000 | 0.275 |
|  | Non-Completers | 170 | 51.93 (11.96) |  |  |  |  |

Independent t-test results comparing KOOS-PS, VR-12 PCS, and VR-12 MCS scores at admission (T0) and discharge (T1) for the TKA sub-sample.

**Table S6.** Comparison of mean age between completers and non-completers (TKA).

| **Characteristic** | **Group** | ***n*** | **M (SD)** | **M_diff_** | ***t*** | **df** | ***p*** |
| --- | --- | --- | --- | --- | --- | --- | --- |
| **Age** | **Completers** | **832** | **67.02 (9.63)** | **−1.43** | **−1.972** | **1063** | **0.049** |
|  | **Non-Completers** | **233** | **68.45 (10.41)** |  |  |  |  |

Independent t-test results for age (years) between the completer and non-completer groups in the TKA sub-sample.

**Table S7.** Comparison of categorical socio-demographic, clinical, and rehabilitation characteristics between completers and non-completers (TKA).

| **Characteristic** | **Category** | **Completers** | | **Non-Completers** | | **Χ²** | **df** | ***p*** |
| --- | --- | --- | --- | --- | --- | --- | --- | --- |
|  |  | ***n*** | **%** | ***n*** | **%** |  |  |  |
| Sex | Female | 508 | 61.1 | 145 | 59.9 | 0.102 | 1 | 0.749 |
|  | Male | 324 | 38.9 | 97 | 40.1 |  |  |  |
| Only German nationality | No | 15 | 1.8 | 4 | 1.7 | 0.016 | 1 | 0.901 |
|  | Yes | 807 | 98.2 | 231 | 98.3 |  |  |  |
| Marital status | Single | 30 | 3.6 | 10 | 4.2 | 5.233 | 5 | 0.388 |
|  | Married | 562 | 67.8 | 157 | 65.7 |  |  |  |
|  | Seperated | 9 | 1.1 | 2 | 0.8 |  |  |  |
|  | Divorced | 79 | 9.5 | 15 | 6.3 |  |  |  |
|  | Widowed | 145 | 17.5 | 53 | 22.2 |  |  |  |
|  | Remarried | 4 | 0.5 | 2 | 0.8 |  |  |  |
| Current committed relationship | No | 166 | 25.7 | 32 | 21.6 | 1.049 | 1 | 0.306 |
|  | Yes | 481 | 74.3 | 116 | 78.4 |  |  |  |
| Highest school degree | In school (enrolled) | 0 | 0.0 | 1 | 0.4 | 5.734 | 6 | 0.454 |
|  | No degree | 14 | 1.7 | 6 | 2.7 |  |  |  |
|  | Special ed. degree | 2 | 0.2 | 1 | 0.4 |  |  |  |
|  | Basic secondary | 413 | 50.7 | 108 | 48.4 |  |  |  |
|  | Intermediate secondary | 249 | 30.6 | 70 | 31.4 |  |  |  |
|  | High school/ Academic secondary | 118 | 14.5 | 30 | 13.5 |  |  |  |
|  | Other | 18 | 2.2 | 7 | 3.1 |  |  |  |
| **Current employment/ income status** | **Employed, full-time** | **155** | **18.6** | **24** | **11.4** | **45.059** | **11** | **<0.001** |
|  | **Employed, part-time** | **86** | **10.3** | **12** | **5.7** |  |  |  |
|  | Marginally employed | 1 | 0.1 | 2 | 1.0 |  |  |  |
|  | Unpaid family worker | 5 | 0.6 | 1 | 0.5 |  |  |  |
|  | Homemaker | 48 | 5.8 | 24 | 11.4 |  |  |  |
|  | Unemployed (registered) | 32 | 3.8 | 4 | 1.9 |  |  |  |
|  | Permanent disability | 23 | 2.8 | 8 | 3.8 |  |  |  |
|  | Temporary disability | 8 | 1.0 | 1 | 0.5 |  |  |  |
|  | Retired | 397 | 47.7 | 104 | 49.5 |  |  |  |
|  | Survivor's benefits | 73 | 8.8 | 23 | 11.0 |  |  |  |
|  | Other not in labor force | 4 | 0.5 | 2 | 1.0 |  |  |  |
|  | Unknown/not specified | 0 | 0.0 | 5 | 2.4 |  |  |  |
| **Work incapacity at admission** | **No** | **560** | **73.3** | **161** | **85.2** | **11.624** | **1** | **<0.001** |
|  | **Yes** | **204** | **26.7** | **28** | **14.8** |  |  |  |
| Pending disability claim | No | 562 | 96.1 | 124 | 96.1 | 0.001 | 1 | 0.976 |
|  | Yes | 23 | 3.9 | 5 | 3.9 |  |  |  |
| **Setting of rehabilitation** | **Inpatient** | **817** | **98.6** | **275** | **96.2** | **6.056** | **1** | **0.014** |
|  | **Day-care** | **12** | **1.4** | **11** | **3.8** |  |  |  |
| Standard rehabilitation in an expedited procedure | No | 777 | 93.4 | 201 | 92.2 | 0.381 | 1 | 0.537 |
|  | Yes | 55 | 6.6 | 17 | 7.8 |  |  |  |
| Side of surgery | Left | 401 | 49.2 | 130 | 45.3 | 2.965 | 2 | 0.227 |
|  | Right | 390 | 47.9 | 152 | 53.0 |  |  |  |
|  | Both sides | 24 | 2.9 | 5 | 1.7 |  |  |  |
| More than five somatic diagnoses | No | 642 | 77.2 | 200 | 80.3 | 1.110 | 1 | 0.292 |
|  | Yes | 190 | 22.8 | 49 | 19.7 |  |  |  |
| **One or more mental disorders** | **No** | **776** | **93.3** | **294** | **96.4** | **3.928** | **1** | **0.047** |
|  | **Yes** | **56** | **6.7** | **11** | **3.6** |  |  |  |

Chi-square test results for sex, citizenship, marital status, education level, employment status, work incapacity, disability claims, rehabilitation setting, surgery side, and comorbidity for the TKA sub-sample.

**Table S8.** Comparison of ProQI scores between high and low data completeness facilities (TKA).

| **Outcome** | **Patients in facilities with Completion rate CR ≥ 75% vs. CR < 75%** | ***n*** | **M (SD)** | **M_diff_** | ***t*** | **df** | ***p*** |
| --- | --- | --- | --- | --- | --- | --- | --- |
| ProQI TKA ^c^ | CR ≥ 75% ^a^ | 533 | 90.23 (23.96) | 0.80 | 0.487 | 823 | 0.627 |
|  | CR < 75% ^b^ | 292 | 89.43 (19.98) |  |  |  |  |

Independent t-test comparing the Patient-Reported Outcome Quality Index (ProQI) between facilities with completion rates ≥ 75% and those with < 75% for TKA.

a: Facilities A, E, F, G, H, I, K, N, O, P, Q, R, V.

b: Facilities B, D, L, M, S, T, U, W.

c: Due to insufficient case numbers (*n* < 15) for facilities C & J, no ProQI could be calculated.
